# Perturbation of Cellular Redox Homeostasis Dictates Divergent Effects of Polybutyl Cyanoacrylate (PBCA) Nanoparticles on Autophagy

**DOI:** 10.3390/cells10123432

**Published:** 2021-12-06

**Authors:** Tonje Sønstevold, Nikolai Engedal, Maria Lyngaas Torgersen

**Affiliations:** 1 Department of Molecular Cell Biology, Institute for Cancer Research, Oslo University Hospital, Montebello, N-0379 Oslo, Norway; tonje.sonstevold@rr-research.no; 2 Department of Tumor Biology, Institute for Cancer Research, Oslo University Hospital, Montebello, N-0379 Oslo, Norway; k.n.engedal@rr-research.no

**Keywords:** autophagy, oxidative stress, nanoparticle, poly(alkyl cyanoacrylate), p38

## Abstract

Nanoparticles (NPs) are used in our everyday life, including as drug delivery vehicles. However, the effects of NPs at the cellular level and their impacts on autophagy are poorly understood. Here, we demonstrate that the NP drug delivery vehicle poly(butyl cyanoacrylate) (PBCA) perturbs redox homeostasis in human epithelial cells, and that the degree of redox perturbation dictates divergent effects of PBCA on autophagy. Specifically, PBCA promoted functional autophagy at low concentrations, whereas it inhibited autophagy at high concentrations. Both effects were completely abolished by the antioxidant N-acetyl cysteine (NAC). High concentrations of PBCA inhibited MAP1LC3B/GABARAP lipidation and LC3 flux, and blocked bulk autophagic cargo flux induced by mTOR inhibition. These effects were mimicked by the redox regulator H_2_O_2_. In contrast, low concentrations of PBCA enhanced bulk autophagic cargo flux in a Vps34-, ULK1/2- and ATG13-dependent manner, yet interestingly, without an accompanying increase in LC3 lipidation or flux. PBCA activated MAP kinase signaling cascades in a redox-dependent manner, and interference with individual signaling components revealed that the autophagy-stimulating effect of PBCA required the action of the JNK and p38–MK2 pathways, whose activities converged on the pro-autophagic protein Beclin-1. Collectively, our results reveal that PBCA exerts a dual effect on autophagy depending on the severity of the NP insult and the resulting perturbation of redox homeostasis. Such a dual autophagy-modifying effect may be of general relevance for redox-perturbing NPs and have important implications in nanomedicine.

## 1. Introduction

Nanotechnology offers great possibilities in medicine, for instance, by bridging the biological and physical barriers of drug delivery. The biocompatible, degradable poly(alkyl cyanoacrylate) (PACA) polymer nanoparticles (NPs), including poly(butyl cyanoacrylate) (PBCA), can efficiently encapsulate bioactive molecules and have been extensively studied for the purpose of drug delivery [1,2,3,4]. In order to better understand their in vivo efficacy and safety, fundamental studies on their interaction with biological systems are needed [5,6]. Of significance, it is important to understand how NPs affect the intracellular degradation pathway autophagy, since alterations in this process can modulate the effect of therapeutic drugs in many clinical settings, and may also affect normal tissue and normal physiology [7,8,9].

Upon interaction with cells, a wide variety of NPs have been shown to cause cellular stress, which, in turn, may induce compensatory protective responses, or lead to cytotoxicity if the insult is too severe. Interestingly, alterations in redox balance are often the underlying mechanism of NP-induced cellular effects [10,11,12]. Redox imbalance may be induced by overproduction of reactive oxygen species (ROS) or by depleting the cellular reserve of ROS scavenger molecules, such as reduced glutathione (GSH) [11,13,14]. Intriguingly, however, oxidative stress can have opposing outcomes; high levels are deleterious to cells and may activate programmed cell death [13], whereas low levels of ROS act as important second messengers in a variety of adaptive pathways, leading to cell growth and survival [14,15]. Thus, through affecting cellular redox balance, NP treatment may have a strong impact on cell signaling pathways and stress responses.

Accumulating evidence indicates that oxidative stress affects the cellular stress pathway autophagy [14,16,17,18,19,20]. However, oxidative stress has been reported to initiate both activating and inhibitory signals towards autophagy, and the mechanisms that dictate the net effect of redox imbalance on autophagy in different contexts remain unclear. Autophagy is an evolutionary conserved catabolic process delivering intracellular material to the lysosomes for degradation. There are three main types of autophagy; macroautophagy, microautophagy, and chaperone-mediated autophagy [21]. Macroautophagy is the best characterized and will henceforth be referred to as autophagy. Mechanistically, the autophagy pathway starts by formation of a double-membrane vesicle, the autophagosome, capturing cargo for degradation either in bulk or in a selective manner, and ends when the autophagosome fuses with the lysosomal compartment, forming the autolysosome, where the captured content is degraded and recycled back to the cytosol. This makes autophagy an important cytoprotective pathway removing damaged organelles and protein aggregates, while providing the cell with nutrients and building blocks for vital cellular functions [22]. Consequently, efficient regulation of autophagy is crucial for the cell to adapt to various stresses.

The formation of lipidated forms of MAP1LC3/GABARAP family proteins has long been recognized as a hallmark of autophagy regulation [23,24]. Several NPs have been reported to regulate autophagy using LC3 as a marker [25]. Upon autophagy activation, cytosolic LC3-I is conjugated to the lipid phosphatidylethanolamine on the forming autophagosomal membrane, generating the lipidated form LC3-II in a process referred to as “LC3 lipidation” [24]. LC3-II remains associated to autophagosomes and is degraded after fusion with the lysosome. Thus, elevated levels of LC3-II may be caused not only by initiation of autophagy, but also when autolysosomal degradation is blocked. To distinguish between these scenarios, it is important to monitor LC3 carrier flux, often called “LC3 flux”, by determining the LC3 protein levels in the absence and presence of a lysosomal inhibitor that blocks LC3-II degradation [23]. However, relying solely on LC3 to monitor autophagy is not recommendable, since additional determination of actual cargo flux through the pathway is needed to draw firm conclusions [23].

The current study represents the first of its kind to utilize a whole suite of different functional autophagy assays, in addition to marker proteins such as LC3, to comprehensively assess NP effects on autophagy. We employed PBCA as a model NP, both because it represents a clinically relevant biodegradable drug carrier NP, and because it perturbs redox homeostasis [26], which is a representative feature of many different NPs of varying composition [10,11,12]. Specifically, our main objectives were to: (i) carefully determine the effect of varying concentrations of PBCA NPs on autophagic activity and the autophagy pathway in human cells, and (ii) to understand the role of redox imbalance and oxidative stress signaling in mediating such effects.

We recently observed that high concentrations of PBCA (25 µg/mL) rapidly reduced basal LC3 lipidation in RPE-1 human retinal epithelial cells and decreased basal degradation of long-lived proteins in MDA-MB-231 breast cancer cells within 4 h of treatment, without inducing cytotoxic effects within this time period [26,27]. This suggested an autophagy-inhibitory effect of PBCA NPs. To explore the ability of PBCA to regulate autophagy in more detail, we here aimed to examine the effect of PBCA also under autophagy-inducing conditions, across a broader NP concentration range, and using a variety of cargo-based assays to monitor autophagic activity. Based on our previous studies of concentration-dependent effects of PBCA in human cells [26,27] as well as initial assessments of the effects of PBCA on cell viability, we chose to examine an NP concentration range of 3.12–25 µg/mL and a treatment period of ≤4 h, with the aim of assessing effects of PBCA NPs on autophagy in the absence of cytotoxicity.

## 2. Materials and Methods

### 2.1. Materials

Hoechst 33342, propidium iodide, L-valine, H_2_O_2_, buthionine sulfoximine, doxycycline, Accutase^®^ Cell Detachment Solution, reduced glutathione (GSH), *N*-acetyl cysteine (NAC), SB202190 and SB203580 were from Sigma-Aldrich (St Louis, MO, USA). Torin1 was purchased from R&D Systems (Minneapolis, MN, USA), Bafilomycin A1 (BafA1) from Enzo Life Sciences (Farmingdale, NY, USA), JNK-IN-8 from Calbiochem (Millipore, Merck, Darmstadt, Germany), BIRB 796 from Axon Medchem BV (Groningen, The Netherlands), U0126 and PF-3644022 from Tocris Bioscience (Bio-Techne Ltd., Abingdon, UK), l-[^14^C]valine from Perkin Elmer (Waltham, MA, USA), and MRT68921 HCl and SAR405 from Selleckchem (Houston, TX, USA). The following antibodies were used for immunoblotting: LC3B (#2775), p-S6K (Thr389; #9205), p-4E-BP1 (Thr37/46; #2855), p-ULK1 (Ser757; #6888), p-eIF2α (#3398), p-AMPKα (Thr172; #2535), AMPKα (#2793), p-ACC (Ser79; #3661), ACC (C83B10) (#3676), p-SAPK/JNK (Thr183/Tyr185; #9251), p-c-Jun (Ser73; #9164), p-ERK1/2 (#9106), p-p38 (#9211), p-MAPKAPK2 (p-MK2; #3041), p-Hsp27 (Ser82; #2401), p-Beclin-1 (Ser90; #86455), and p-Bcl-2 (Ser70; #2827) from Cell Signaling Technology (Danvers, MA, USA), Vinculin (sc-59803) from Santa Cruz Biotechnology (Dallas, TX, USA), mKeima-red (M126-3M) and GABARAP (#PM037) from MBL International (Woburn, MA, USA), β-Actin (AC-74) (A5316) from Sigma-Aldrich (St Louis, MO, USA), Peroxidase AffiniPure Goat Anti-Mouse IgG (#115-035-003) and Peroxidase AffiniPure Goat Anti-Rabbit IgG (#111-035-144) from Jackson ImmunoResearch Europe Ltd. (Cambridge, UK).

### 2.2. Nanoparticle Synthesis

PBCA NPs were prepared using the mini-emulsion polymerization method as previously described [1,26,28]. Briefly, PBCA was made by mixing the oil phase, consisting of the monomer butyl cyanoacrylate (2.5 g), a neutral oil (Miglyol 812, 2 wt%), and vanillin (10 wt%), with the aqueous phase consisting of hydrochloric acid (0.1 M, 25 mL) and the PEG-surfactants Kolliphor^®^ HS15 (6 mM) and Pluronic^®^ F68 (2 mM). The oil in water mini-emulsion was made using a tip sonifier (Branson, 50% amplitude, 6 × 30 s with 10 s pauses, on ice). The polymerization was carried out at room temperature overnight. The pH was increased to pH 5 using 0.1 M NaOH and the polymerization continued for 5 h. Surplus of surfactants were removed by extensive dialysis against 1 mM HCl (pH 3). Size, polydispersity index and ζ-potential were determined using dynamic and electrophoretic light scattering (Zetasizer Nano ZS, Malvern Instruments, Malvern, UK) in 0.01 M phosphate buffer, pH 7. The reported NP mean diameter (nm) is the *Z*-average. The resulting PBCA NPs were 165 ± 36 nm, with a polydispersity index of 0.15 ± 0.05 and slightly negatively charged with a zeta-potential of −3.2 ± 0.05 mV. POCA NPs produced in the same manner were used as a positive control for caspase activation [26]. NP concentrations were determined as previously described [1].

### 2.3. Cells and Treatments

The human cell lines RPE-1, U2OS and HEK293 were obtained from ATCC, Manassas, VA, USA and cultured in DMEM/F12 (RPE-1) or DMEM (U2OS and HEK293) supplemented with 10% fetal bovine serum (FBS), 100 U/mL penicillin and 100 µg/mL streptomycin (herein referred to as complete medium, CM) (all from Sigma-Aldrich, St. Louis, MO, USA) at 37 °C and 5% CO_2_. The cells have been authenticated and regularly tested for mycoplasma contamination. Depending on the assay, cells were seeded one or two days prior to the experiments. Cells were starved for amino acids by incubation in Earle’s balanced salt solution (EBSS, #24010043, Thermo Fisher Scientific, Waltham, MA, USA) supplemented with 10% dialyzed FBS (EBSS/FBS, dialysis tube cutoff 3.5 kDa). For studies using inhibitors or antioxidants, the cells were pre-treated for either 1.5 h (BIRB 796 and U0126, both 10 µM), 1 h (NAC, 3 mM; GSH, 10 mM), or 30 min (SB203580, SB202190 and JNK-IN-8, 3 µM; PF-3644022, 2.5 µM) before addition of PBCA. To assess cell viability after PBCA treatment, cellular ATP was measured as an indicator of metabolically active cells using CellTiter-Glo^®^ Luminescent Cell Viability Assay (#G7571, Promega, Madison, WI, USA). Luminescence was measured by a Synergy2 plate reader (BioTek Instruments Inc., Winooski, VT, USA).

### 2.4. Immunoblotting

Treated cells were washed with cold PBS and lysed in 1.1× NuPAGE LDS Sample buffer (#N0008, Invitrogen, Carlsbad, CA, USA) containing 110 mM DTT. The lysates were boiled (10 min, 98 °C) and sonicated (10 s, 25% amplitude) to reduce viscosity. All lysates were separated by 4–20% SDS-PAGE and transferred to a PVDF membrane. The membrane was dried and incubated with the indicated primary antibodies in 5% bovine serum albumin overnight at 4 °C, followed by washing and 35 min incubation with HRP-conjugated secondary antibodies at room temperature and detection with SuperSignal West Dura Extended Duration Substrate (Thermo Fisher Scientific, Waltham, MA, USA) in a ChemiDoc Imaging System (Bio-Rad Laboratories, Hercules, CA, USA). The signal intensities were quantified by the Image Lab Software (Bio-Rad Laboratories, Hercules, CA, USA).

### 2.5. Microscopy

Cells were seeded on glass coverslips for experimental treatment, washed once with PBS and fixed in 10% formalin for 15 min. Permeabilization was performed by 5 min incubation with PBS containing 0.1% Triton X-100 and cells were blocked by 30 min incubation with PBS containing 5% FBS. Cells were stained for 30 min with an anti-LC3 polyclonal antibody (#PM036, MBL International, Woburn, MA, USA) diluted 1:500 in PBS containing 5% FBS, washed three times and further stained 30 min with Alexa488-conjugated donkey anti-rabbit (#711-545-152, Jackson Immunoresearch Europe Ltd., Cambridge, UK) diluted 1:500 in PBS containing 5% FBS. Cells were washed and nuclei stained with 5 µg/mL Hoechst for 10 min before coverslips were mounted in Mowiol^®^ 4-88 (#81381, Sigma-Aldrich, St Louis, MO, USA). The cells were imaged using an Olympus ScanR illumination system with an UPLSAPO 40× objective. The ScanR analysis software was used for background correction and automatic image analysis from a large number of wide-field images. Identical imaging and analysis settings were applied for all treatment conditions. Cells were identified and segmented based on the Hoechst nuclear stain, and the total fluorescent intensity of LC3 in segmented puncta within each cell was measured.

### 2.6. Inducible mKeima Assay System

#### 2.6.1. Generation of Stable Cell Lines

By lentiviral transduction we generated RPE-1 cells stably expressing the following doxycycline-inducible mKeima probes: free, cytosolic mKeima as a bulk autophagy cargo, LDHB-mKeima, where mKeima is fused to the cytosolic enzyme lactate dehydrogenase B (LDHB), which is sequestered exclusively by autophagy for degradation [29] and used as a bulk cargo reporter protein [30,31,32] (Supplementary Figure S4), and mKeima-LC3B, where mKeima is fused to the autophagic marker LC3B (Supplementary Figure S6). The pLVX-TetOne-Puro plasmids (Clontech, Takara Bio Inc., San Jose, CA, USA) encoding free mKeima or the mKeima fusion proteins were kind gifts from Dr. Lisa Frankel (Danish Cancer Society Research Center, Copenhagen, Denmark). Lentivirus particles were packaged using the plasmids psPAX2 (Addgene #12260) and pMD2.G (Addgene #12259) as previously described [33]. Cells were then transduced with low virus titers (multiplicity of infection (m.o.i.) < 1) and stable cell pools were generated by selection with puromycin (5–10 μg/mL). Approximately 10–20% of the cells were transduced, as judged by the percentage of cells being selected during puromycin treatment.

#### 2.6.2. Detection of mKeima Processing

Monomeric Keima (mKeima) is a pH-responsive fluorescent protein resistant to lysosomal proteases, thus providing a cumulative readout of autophagic activity as it stably accumulates and undergoes a change in chromophore resting charge state upon trafficking to the acidic environment of lysosomes (pH~4.5) [34]. For optimal induction of mKeima expression, the stable RPE-1 cells were treated for 24–48 h with 100 ng/mL doxycycline. Before further treatments, the cells were washed twice to remove doxycycline. The cells were subsequently treated as described in each figure legend, and autophagic processing and flux of the mKeima fusion proteins was detected by immunoblotting and flow cytometry, respectively. Accumulation of the free, undegradable mKeima-part of the fusion proteins was detected by immunoblotting against mKeima after direct lysis of the cells in 1.1× NuPAGE LDS sample buffer as described in detail under “Immunoblotting”.

For flow cytometry, treated cells were detached by Accutase^®^ Cell Detachment Solution, washed and resuspended in PBS containing 1% FBS, 1 mM EDTA, 25 mM Hepes (pH 7.3–7.5) for analysis on a BD LSR II Flow Cytometer (BD Biosciences, San Jose, CA, USA) connected to the BD FACSDiva™ software (BD Biosciences, San Jose, CA, USA). Since mKeima has a bimodal excitation spectrum peaking at 440 and 586 nm, corresponding to the neutral and ionized states of the chromophore’s phenolic hydroxyl moiety [35], autophagic flux was measured as the ratio of the median fluorescent intensity of mKeima excited by 561 nm (45 mW) laser divided by mKeima excited by 407 nm (100 mW) laser, with a 610/20 bandpass filter and a 600 nm long pass dichroic filter in both cases. By using the FlowJo™ software (BD Biosciences, San Jose, CA, USA), the derived ratio of 561/407 nm signal intensity per cell was obtained, and the median value of these cellular ratios were compared between treatments. The bimodal excitation spectrum of mKeima renders this assay incompatible with various live/dead stains without compensation. Thus, the samples were first read for autophagic flux measurements, thereafter propidium iodide was added and the sample read again for setting of live/dead gates (see Supplementary Figure S4C for detailed gating strategy).

### 2.7. Long-Lived Protein Degradation Assay (LLPD)

The assay is based on a pulse-chase labelling approach to study protein turnover and was performed as described in [36], with some modifications. Cells were incubated for 24 h with 0.135 μCi/mL l-[^14^C]valine in RPMI complete medium, followed by a 3 h chase in RPMI complete medium supplemented with 10 mM non-radioactive L-valine to allow degradation of short-lived proteins. Cells were further washed and subjected to experimental treatments to initiate a 4 h probing of long-lived protein degradation. Cellular protein was precipitated with 10% (vol/vol final concentration) trichloroacetic acid, and percent degradation was assessed as the acid-soluble radioactivity divided by the total radioactivity. To enable calculation of the autophagic fraction of LLPD, the assay was performed in the absence or presence of the Vps34 inhibitor SAR405, which inhibits autophagosome formation and thus the autophagic–lysosomal degradation pathway.

### 2.8. Autophagic LDH Sequestration Assay

The assay was performed as described in detail in [30], with minor alterations. Briefly, LDH sequestration was assessed after 3 h treatment in the presence of BafA1, to prevent autolysosomal LDH degradation. Untreated wells defined background levels, and BafA1-only wells defined basal levels of sequestered LDH. Cells were harvested by Accutase^®^ Cell Detachment Solution at room temperature and resuspended in 10% sucrose. The plasma membrane of the cells was selectively disrupted using a custom-made electroporator (2000 V and 1.2 μF in a 1 cm × 1 cm × 5 cm electrode chamber). An amount of 150 μL total cell disruptates was mixed with 450 μL resuspension buffer containing 1.33% Triton X-405 at room temperature on a shaker. Then, 600 μL resuspension buffer containing 1% Triton X-405 was added to the sedimented samples. The amount of LDH in each sample was quantified as the decline in NADH absorbance at 340 nm in an enzymatic reaction with 0.6 mM pyruvate and 0.36 mM NADH in 65 mM imidazole (pH 7.5), using a multianalyzer (MaxMat PL-II, Erba Diagnostics, Mannheim, Germany), and the percentage sedimentable LDH to total LDH was calculated. LDH sequestration activity was calculated as percent sedimentable LDH in experimentally treated cells minus percent sedimentable LDH in untreated control cells (background) divided by treatment time with BafA1.

### 2.9. Detection of Reactive Oxygen Species (ROS)

Intracellular ROS accumulation was detected by the chloromethyl derivative of the fluorogenic dye 2′,7′-dichlorodihydrofluorescein diacetate (CM-H_2_DCFDA) (#C6827, Invitrogen, Carlsbad, CA, USA) according to the manufacturer’s guidelines. Cells were rinsed in DMEM/F12 without phenol red and FBS, before pre-incubation with the dye (10 µM, 45 min) in the same medium. Subsequently, cells were washed twice in CM and treated 4 h with PBCA at increasing concentrations. Cells were detached by Accutase^®^ Cell Detachment Solution, washed and resuspended in PBS containing 1% FBS, 1 mM EDTA, 25 mM Hepes (pH 7.3–7.5) for analysis on a BD LSR II Flow Cytometer (BD Biosciences, San Jose, CA, USA) connected to the BD FACSDiva™ software (BD Biosciences, San Jose, CA, USA). Hoechst was added as live/dead stain using a 405 nm (100 mW) laser with a 450/50 nm bandpass filter. ROS was measured as the median fluorescent intensity of live cells using 488 nm (100 mW) laser with a 525/30 nm bandpass filter and a 505 nm long pass dichroic filter. The data were analyzed by FlowJo™ software (BD Biosciences, San Jose, CA, USA).

### 2.10. Glutathione Measurement (GSH)

Intracellular levels of GSH were measured by o-phthalaldehyde (#79760 Sigma-Aldrich, St Louis, MO, USA) as previously described [37]. Treated cells (1.2 × 10^6^ cells in 5 cm dish) were harvested by Accutase^®^ Cell Detachment Solution, washed with PBS, lysed in PBS containing 0.5% NP40 and cOmplete™ protease inhibitor cocktail (#5056489001, Roche Diagnostics, Mannheim, Germany), and precipitated with 5% trichloroacetic acid on ice for 10 min. Subsequently, the cell lysates were centrifuged (10 min, 12,000× *g*) and the supernatants were incubated for 10 min at room temperature with o-phthalaldehyde (1 mg/mL final concentration) in phosphate buffer (pH 8.0). The fluorescence intensity of the formed complex was measured by a Synergy2 plate reader (BioTek) with excitation and emission wavelengths of 360 nm and 460 nm, respectively. Inhibition of glutathione synthesis by buthionine sulphoximine (100 µM, 5 h) was used as a positive control for GSH depletion. The GSH content was normalized to the protein content of each lysate, as determined by the BCA assay (Thermo Fisher Scientific, Waltham, MA, USA).

### 2.11. Statistical Analysis

Mean values ± standard error of the mean (SEM) were calculated for each condition. The statistical significance of the differences was determined by two-tailed paired or unpaired Student’s *t*-test, with equal or unequal variances, as appropriate; *, *p* < 0.05; **, *p* < 0.01; ***, *p* < 0.001.

## 3. Results

### 3.1. PBCA NPs Trigger Concentration-Dependent, Divergent Effects on Autophagy

**Figure 1:**
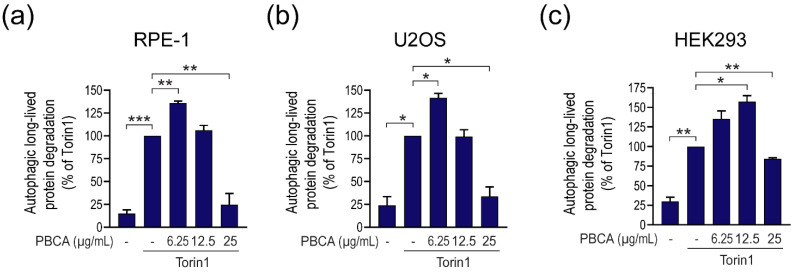
PBCA stimulates degradation of long-lived proteins at low NP concentrations and inhibits degradation at high concentrations. RPE-1 (**a**), U2OS (**b**) and HEK293 (**c**) cells were radiolabeled with [^14^C]valine for 1 d and chased for 3 h. Subsequently, cells were treated with Torin1 (50 nM) and the indicated concentrations of PBCA in the absence or presence of the Vps34-inhibitor SAR405 (10 µM) for 4 h. The autophagic fraction of degradation of long-lived proteins was determined as described in Materials and methods and Supplementary Figure S1. All graphs show mean values ± SEM quantified from at least three independent experiments. *, *p* < 0.05; **, *p* < 0.01; ***, *p* < 0.001.

In order to determine how PBCA influences autophagy in RPE-1 cells upon classical autophagy induction through inhibition of the master autophagy regulator mTOR, we treated cells with the mTOR-inhibitor Torin1 in the absence or presence of increasing concentrations of PBCA for 4 h, and determined autophagic degradation by the long-lived protein degradation (LLPD) assay, a standard method to monitor autophagic activity [23,36]. To specifically determine the autophagic fraction of LLPD, the experiments were performed in the absence or presence of the Vps34 inhibitor SAR405, as detailed in Supplementary Figure S1. Strikingly, Torin1-induced autophagic LLPD was almost completely abolished when cells were co-treated with 25 µg/mL PBCA (Figure 1a), indicating a strong anti-autophagic effect of PBCA at high concentrations. However, at lower concentrations (6.25 µg/mL), PBCA significantly *increased* autophagic degradation activity (Figure 1a). Together, this suggests a biphasic, divergent effect of PBCA on autophagy, depending on the NP concentration. This phenomenon was not restricted to RPE-1 cells, as we observed the same biphasic effect of PBCA also in the human osteosarcoma cell line U2OS and in the human embryonic kidney cell line HEK293 (Figure 1b,c).

**Figure 2:**
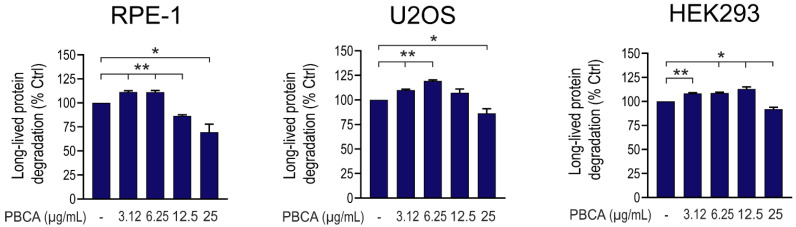
PBCA induces a divergent effect on basal long-lived protein degradation in epithelial cells. RPE-1, U2OS and HEK293 cells were radiolabeled with [^14^C]valine for 1 d, chased for 3 h, and subsequently treated with the indicated concentrations of PBCA for 4 h. The graphs show mean values ± SEM from three independent experiments. The asterisks denote statistical significances compared to non-treated control. *, *p* < 0.05; **, *p* < 0.01.

**Figure 3:**
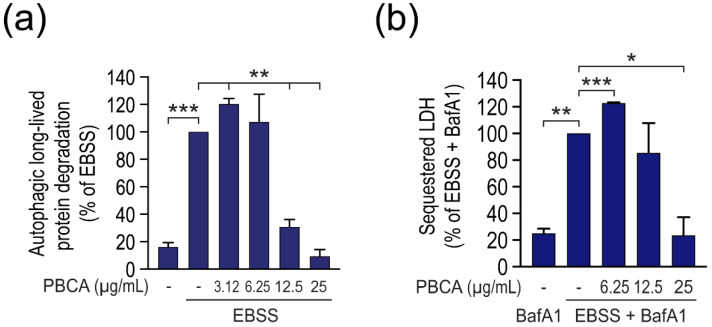
PBCA alters autophagic activity in a divergent manner under amino acid starvation conditions. (**a**) RPE-1 cells were radiolabeled with [^14^C]valine for 1 d and chased for 3 h. Subsequently, cells were starved in EBSS/FBS (“EBSS”) and treated with PBCA at the indicated concentrations in the absence or presence of SAR405 (10 µM) for 4 h, followed by determination of the autophagic fraction of long-lived protein degradation. (**b**) RPE-1 cells were maintained in nutrient-rich medium, or starved in EBSS/FBS (“EBSS”), and treated with the indicated concentrations of PBCA in the presence of BafA1 (100 nM) for 3 h. The cells were harvested and the amount of sequestered LDH was measured as detailed in Materials and Methods, and normalized to the sequestration activity in the EBSS + BafA1 condition (set to 100). Both graphs show mean values ± SEM quantified from at least three independent experiments. The asterisks denote statistical significances compared to treatment with EBSS alone (**a**), or EBSS + BafA1 alone (**b**). *, *p* < 0.05; **, *p* < 0.01; ***, *p* < 0.001.

The same divergent effect of PBCA on long-lived protein degradation was observed under basal, nutrient-rich conditions in all three cell types (Figure 2), as well as when autophagy was induced by amino acid starvation instead of Torin1 (Figure 3a).

Moreover, the biphasic effect of PBCA on autophagy was confirmed when using another classical method to measure bulk autophagic activity, the “lactate dehydrogenase (LDH) sequestration assay” [23,30] (Figure 3b, and see Supplementary Figure S2 and its legend for a description of the assay).

Importantly, the observed effects of PBCA on autophagy were unrelated to any cytotoxic effects of the NPs, as the autophagy modulation was observed already within 4 h of treatment, whereas PBCA-induced cytotoxicity occurs much later and/or requires much higher concentrations of PBCA. Thus, even when RPE-1 cells were treated with as much as 100 µg/mL PBCA for 4 h or 8 h, there was little change in cell viability as determined by quantification of ATP as an indicator of metabolically active cells (Supplementary Figure S3A). Moreover, there were no signs of any caspase activation within 4 h of treatment with 25 µg/mL PBCA (Supplementary Figure S3B), indicating absence of apoptosis initiation within this time period. U2OS and HEK293 cells also tolerated PBCA very well within shorter time points, and even after treatment with 25 µg/mL PBCA for as long as 24 h, cell viability remained high in both cell lines (>75% viability compared to control treatment; Supplementary Figure S3C,D). These data are in line with cell viability data from six additional human cell lines [26].

To examine the activating and inhibitory effects of PBCA in more detail, we took advantage of a versatile method to monitor autophagic flux of both cargo and membrane marker proteins (such as LC3), which is based on the fluorescent coral protein mKeima [31,32,34]. We focused on the effect of PBCA in RPE-1 cells treated with Torin1, since we observed the strongest activating and inhibitory effects of PBCA on autophagy under those conditions.

**Figure 4:**
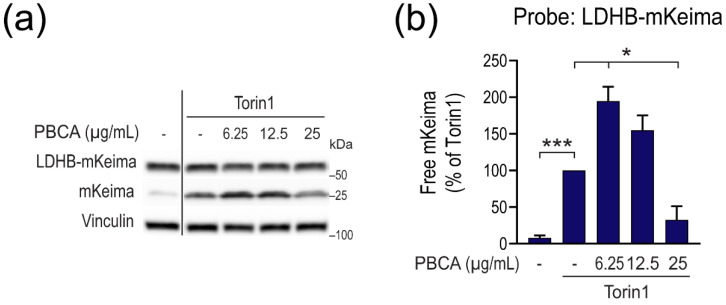
PBCA stimulates autophagic cargo processing at low NP concentrations and inhibits autophagy at high concentrations. RPE-1 cells with doxycycline-controlled expression of LDHB-mKeima were induced with 100 ng/mL doxycycline for 2 d, washed, and treated with PBCA at the indicated concentrations in the absence or presence of Torin1 (50 nM) for 4 h. (**a**) Cell lysates were prepared for immunoblotting and the blots were probed with the indicated antibodies. The solid line indicates a cut of the blot for illustration purposes. The position of molecular weight markers is indicated at the right-hand side of the blots. (**b**) The relative amounts of free mKeima were quantified. The graph shows mean values ± SEM quantified from at least three independent experiments. The asterisks denote the statistical significances compared to treatment with Torin1 alone. *, *p* < 0.05; ***, *p* < 0.001.

We first employed a variant of the mKeima-based assays which, similarly to the LLPD and LDH sequestration assays, measures bulk autophagic cargo flux [31,32]. This assay, referred to as the “LDHB-mKeima processing assay”, is based on expressing a fusion protein consisting of the cytosolic protein LDHB fused to mKeima as a cargo probe. During general autophagy, cytosol, including the LDHB-mKeima probe, is taken up into autophagosomes along with other cargo, and subsequently autophagosomes fuse with lysosomes for degradation of the sequestered cargo. As opposed to LDHB, mKeima is resistant to autolysosomal degradation [34], and thus, autophagic flux can be tracked by immunoblot detection of the free mKeima that is produced upon LDHB-mKeima processing [31,32]. We generated RPE-1 cells that express LDHB-mKeima under the control of doxycycline (Supplementary Figure S4) and subjected them to Torin1-treatment in the absence or presence of PBCA. As expected, and confirming the results obtained with the LLPD and LDH sequestration assays, 6.25 µg/mL PBCA significantly increased the production of free mKeima, whereas 25 µg/mL PBCA strongly inhibited LDHB-mKeima processing (Figure 4).

In addition to immunoblotting for the generation of free mKeima, the flux of LDHB-mKeima to the autolysosomal compartment can be monitored by the fluorescence signal ratio obtained from cells excited with the two lasers, 561 nm (which predominantly excites mKeima in acidic compartments, e.g., autolysosomes) and 407 nm (which predominantly excites mKeima in non-acidic compartments, e.g., cytosol or autophagosomes) [31,32,34] (Supplementary Figure S4C). Analyses of Torin1-treated LDHB-mKeima-expressing RPE-1 cells by flow cytometry revealed a consistent and significant *increase* in 561/407 nm signal ratio in cells treated with low concentrations of PBCA (3.12–12.5 μg/mL) and a *decrease* at high PBCA concentrations (25 μg/mL) (Supplementary Figure S5A,B). This again indicates that low PBCA concentrations enhance autophagic flux whereas high concentrations do the opposite. To further validate these findings, we performed corresponding experiments in cells expressing mKeima, which like LDHB-mKeima is a soluble cytosolic protein. As shown in Supplementary Figure S5C,D, we obtained essentially identical results when using mKeima as a probe in place of LDHB-mKeima.

Together, these results support the findings observed with the classical LLPD and LDH sequestration assays and enabled us to firmly establish that PBCA stimulates autophagy at low concentrations, whereas it blocks autophagy at high concentrations. Moreover, the comparative results obtained with the different methods showed that we could use mKeima-based assays to reliably monitor the effects of PBCA on autophagy.

### 3.2. High Concentrations of PBCA Inhibit Autophagic LC3 Flux and LC3 Lipidation, Whereas Low Concentrations Activate Canonical, Yet LC3 Flux-Independent Autophagy

An important question to answer was whether the divergent effects of PBCA on autophagic cargo flux and cargo degradation were accompanied by similar effects on the flux and degradation of the autophagosome marker LC3. To this end, we established RPE-1 cells expressing mKeima-LC3B (Supplementary Figure S6A,B), and assessed the effect of PBCA on free mKeima production by immunoblotting.

**Figure 5:**
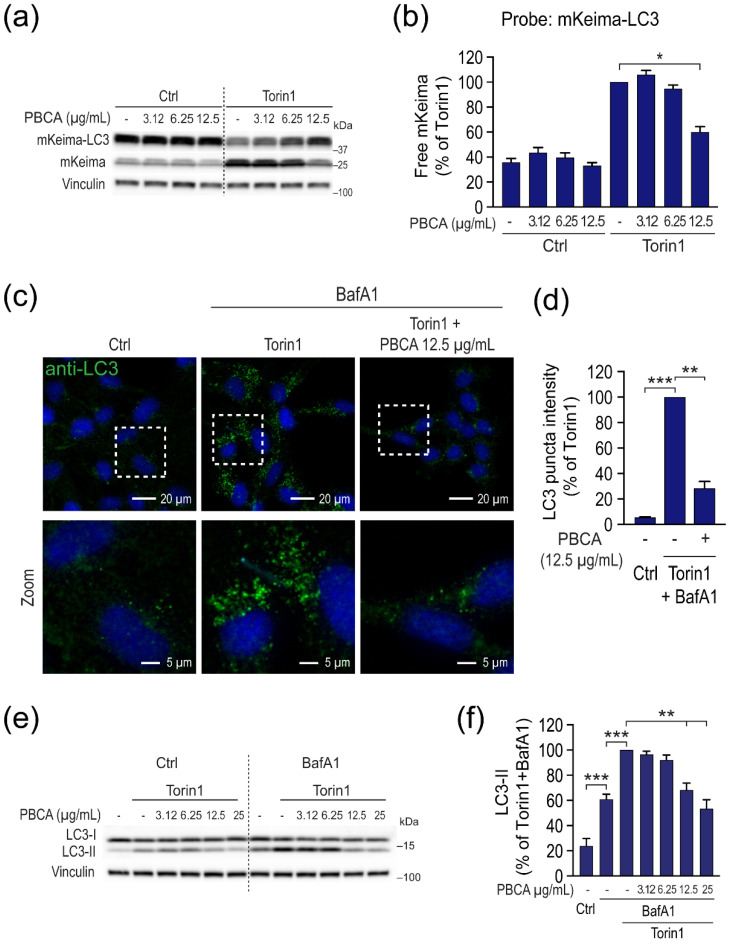
PBCA inhibits LC3 flux and puncta formation. (**a**,**b**) RPE-1 cells with doxycycline-controlled expression of mKeima-LC3 were induced with 100 ng/mL doxycycline for 1 d, washed, and treated with PBCA at the indicated concentrations in the absence or presence of Torin1 (50 nM) for 4 h. Cell lysates were prepared for immunoblotting, and the relative amounts of free mKeima were quantified. (**c**,**d**) RPE-1 cells were treated with PBCA (12.5 µg/mL) and Torin1 (50 nM) for 1 h in the presence of BafA1 (100 nM). Cells were fixed and stained with an anti-LC3 antibody. Nuclei were stained with Hoechst, and images were acquired by confocal microscopy (**c**). The relative levels of LC3 puncta intensity were quantified (**d**). (**e**,**f**) RPE-1 cells were treated with PBCA (indicated concentrations) and Torin1 (50 nM) for 1 h in the absence and presence of BafA1 (100 nM). Cell lysates were prepared for immunoblotting and the blots were probed with the indicated antibodies (**e**). The relative amounts of LC3-II were quantified (**f**). All immunoblot samples were compared side by side on the same gel; the vertical dashed lines are drawn to simplify comparison between treatments. All graphs show mean values ± SEM quantified from at least three independent experiments. *, *p* < 0.05; **, *p* < 0.01; ***, *p* < 0.001.

Torin1-induced flux of mKeima-LC3 was completely abolished by co-treatment with SAR405, which inhibits the autophagy-essential lipid kinase Vps34, or by siRNA-mediated depletion of the crucial autophagy machinery components ULK1 and ULK2 (Supplementary Figure S6C–E), confirming that the expressed mKeima-LC3 is functional and reliably monitors autophagy-dependent LC3 flux. The strong inhibitory effect of 12.5 µg/mL PBCA on Torin1-induced LC3 flux was verified by fluorescent staining of endogenous LC3 puncta (Figure 5c,d), and by immunoblotting of endogenous LC3 (Figure 5e,f; Supplementary Figure S7A) in the absence or presence of Bafilomycin A1 (BafA1), which is used to block LC3-II degradation and thus reveal changes in LC3-II levels independently of its degradation [23]. The strong ability of high PBCA concentrations to reduce the levels of lipidated LC3 (LC3-II) (Figure 5e,f) suggests that PBCA blocks autophagy by interfering with the ATG8 lipidation machinery. Indeed, lipidation of another mammalian ATG8 homolog, GABARAP, was also inhibited by PBCA in a concentration-dependent manner, and both in the absence and presence of BafA1 (Supplementary Figure S7B,C).

Taken together, whereas high concentrations of PBCA block both autophagic cargo and LC3 flux, low concentrations of PBCA enhance autophagic cargo flux and degradation without a concomitant increase in LC3 lipidation, puncta formation, or turnover. The latter implies that PBCA activates an LC3 flux-independent form of autophagy at low concentrations. This unexpected observation prompted us to further evaluate the nature of PBCA-induced autophagy, specifically as to whether it depends on canonical autophagy machinery components, e.g., Vps34, ULK1/2, and ATG13. Indeed, PBCA-mediated enhancement of LDHB-mKeima flux was completely abolished by the Vps34-inhibitor SAR405, by the dual ULK1/2 inhibitor MRT68921, or by siRNA-mediated depletion of ATG13 (Supplementary Figure S8). Thus, we conclude that PBCA activates canonical, yet LC3 flux-independent autophagy. Of note, this effect of PBCA was not mediated via regulation of mTOR, since PBCA did not alter Torin1-mediated inhibition of mTORC1 activity (Supplementary Figure S9A), and PBCA enhanced LDHB-mKeima flux to the same extent even if the Torin1 concentration was increased by 10-fold (Supplementary Figure S9B).

### 3.3. PBCA-Induced Redox Imbalance Is Required for Activation of Autophagy at Low PBCA Concentrations, and Mediates Inhibition of LC3 Lipidation and Autophagy, as Well as Subsequent Cytotoxicity at High PBCA Concentrations

**Figure 6:**
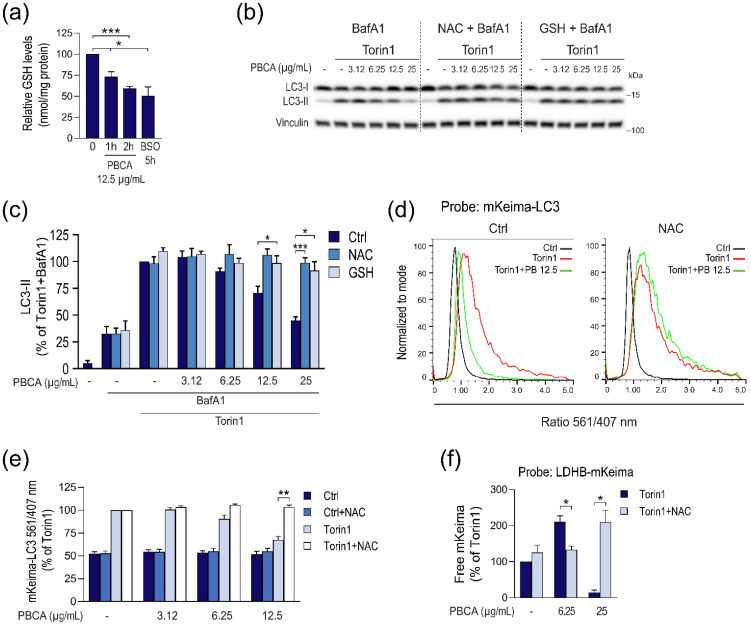
PBCA-mediated effects on autophagy are prevented by NAC and GSH. (**a**) The intracellular concentration of GSH was determined with o-phthalaldehyde in RPE-1 cells treated with PBCA (12.5 µg/mL) for 1 or 2 h. Inhibition of glutathione synthesis by buthionine sulfoximine (BSO, 100 µM) for 5 h was used as a positive control for GSH depletion. (**b**,**c**) RPE-1 cells were pre-treated with NAC (3 mM) or GSH (10 mM) for 1 h before addition of BafA1 (100 nM), Torin1 (50 nM) and PBCA at the indicated concentrations for 1 h. Cell lysates were prepared for immunoblotting and the relative amount of LC3-II was quantified (**c**). In (**b**), the immunoblot samples were compared side by side on the same gel; the vertical dashed lines are included merely to simplify comparison between treatments. (**d**,**e**) RPE-1 cells with inducible expression of mKeima-LC3 were induced for 1 d, washed, and pre-treated with NAC (3 mM) for 1 h before addition of Torin1 (50 nM) and PBCA at the indicated concentrations. The incubation was continued for 4 h before harvesting, and the 561/407 nm fluorescence intensity ratios were determined by flow cytometry. Individual ratio histograms from one representative experiment with PBCA 12.5 µg/mL are shown (**d**), and the relative median values of the 561/407 nm ratios were quantified (**e**). (**f**) RPE-1 cells with inducible expression of LDHB-mKeima were induced for 2 d, washed, and pre-treated with NAC (3 mM) for 1 h before addition of Torin1 (50 nM) and PBCA at the indicated concentrations. The incubation was continued for 4 h before cell lysates were prepared for immunoblotting, and the relative amounts of free mKeima were quantified. All graphs show mean values ± SEM quantified from at least three independent experiments. *, *p* < 0.05; **, *p* < 0.01; ***, *p* < 0.001.

Since we previously demonstrated that redox imbalance is one of the main contributing factors to PBCA-induced cellular stress and cytotoxicity in MDA-MB-231 cells [26], we assessed whether treatment with PBCA leads to redox imbalance also in RPE-1 cells. To that end, we used the fluorescent probe CM-H_2_DCFDA, and monitored cellular ROS levels by flow cytometry. While ROS levels remained seemingly unchanged during the first 2 h of treatment (data not shown), a marked concentration-dependent increase was detected after 4 h (Supplementary Figure S10). Redox imbalance is not only caused by accumulation of ROS, but is often associated with depletion of GSH, one of the cells’ primary antioxidants [38]. We therefore also measured cellular GSH levels after PBCA treatment. Strikingly, treatment with 12.5 µg/mL PBCA led to significant depletion of the cellular GSH reserves already after 1 h (Figure 6a). After 2 h, the GSH level was further reduced, down to a level comparable to that obtained by the positive control buthionine sulphoximine, a γ-glutamyl cysteine synthetase inhibitor [39] (Figure 6a). Collectively, our results indicate that PBCA treatment causes a rapid redox imbalance that leads to a concentration-dependent accumulation of oxidative stress in RPE-1 cells.

Decreased GSH redox buffering capacity may render intracellular protein thiols prone to ROS-mediated oxidation, including inactivating oxidations of LC3 lipidation machinery proteins [18]. We therefore asked whether an excess of antioxidants would rescue PBCA-mediated inhibition of LC3 lipidation. Hence, Torin1-induced LC3 lipidation was assessed in the absence or presence of NAC, a widely used pharmacological antioxidant, or in the presence of excess GSH. To enable assessment of LC3 flux, the cells were co-treated with BafA1 to inhibit autolysosomal LC3 degradation. As expected, Torin1 significantly increased LC3 lipidation under all conditions, and, as observed earlier, high concentrations of PBCA inhibited LC3 lipidation (Figure 6b,c). Interestingly, however, the presence of NAC or GSH completely abolished the inhibitory effect of PBCA (Figure 6b,c). To gain further insight, we tested whether excess antioxidants would rescue also PBCA-mediated inhibition of autolysosomal mKeima-LC3 processing. Indeed, as assessed by both flow cytometry (Figure 6d,e) and immunoblotting (Supplementary Figure S11A,B), co-treatment with NAC completely prevented the inhibitory effect of PBCA.

Having shown that the presence of NAC or excess GSH rescues PBCA-mediated inhibition of LC3 lipidation and flux, we asked whether antioxidants would influence also the divergent effects of PBCA on autophagic cargo flux. For this, we monitored Torin1-induced LDHB-mKeima processing, and assessed potential effects of NAC on the activation of autophagy observed with 6.25 µg/mL PBCA and the inhibition observed with 25 µg/mL PBCA. Strikingly, the presence of NAC completely abolished both the potentiation and the inhibition of LDHB-mKeima processing (Figure 6f).

In summary, adding an excess of antioxidants abrogates the PBCA-mediated inhibition of LC3-lipidation and autophagic cargo degradation observed at high NP concentrations, and prevents the potentiation of Torin1-induced autophagy observed at low NP concentrations.

Although autophagy-inhibitory concentrations of PBCA (25 μg/mL) did not induce any cytotoxicity within the first 4 h of treatment in RPE-1 cells (Supplementary Figure S3A,B), it reduced cell viability by ~45% after 24 h of treatment (Supplementary Figure S12). In contrast, autophagy-inducing PBCA concentrations (6.25 µg/mL) did not diminish cell viability even after 24 h of treatment (Supplementary Figure S12). Excess NAC or GSH effectively abolished the cytotoxicity observed upon 24 h treatment of RPE-1 cells with 25 μg/mL PBCA (Supplementary Figure S12), in line with data obtained in MDA-MB-231 cells [26].

Taken together, our results indicate that high concentrations of PBCA induce a level of oxidative stress that first mediates a block in autophagy, and secondary is required for cell death. At lower concentrations, PBCA NPs induce a lower level of oxidative stress that is essential for upregulation of autophagic activity, which in turn may help in maintaining cellular homeostasis and cell viability.

### 3.4. H_2_O_2_ Inhibits LC3 Lipidation and Autophagic Degradation

**Figure 7:**
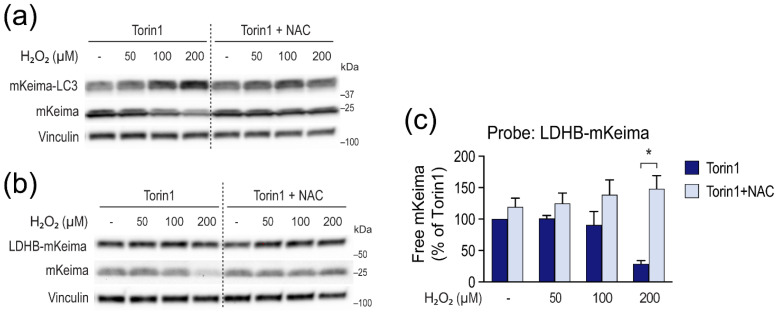
Treatment with H_2_O_2_ inhibits Torin1-induced autophagic carrier and cargo flux. (**a**) RPE-1 cells with inducible expression of mKeima-LC3 were induced for 1 d, washed, and pre-treated with NAC (3 mM) for 1 h before addition of Torin1 (50 nM) and H_2_O_2_ at the indicated concentrations. The incubation was continued for 4 h before cell lysates were prepared for immunoblotting. (**b**,**c**) RPE-1 cells with inducible expression of LDHB-mKeima were induced for 2 d, washed, and pre-treated with NAC (3 mM) for 1 h before addition of Torin1 (50 nM) and H_2_O_2_ at the indicated concentrations. The incubation was continued for 4 h before cell lysates were prepared for immunoblotting (**b**), and the relative amounts of free mKeima were quantified (**c**). The graph shows mean values ± SEM quantified from at least three independent experiments. *, *p* < 0.05. The dashed lines are introduced in the immunoblots for visual purposes only (i.e., the blots were not cut).

We aimed to clarify whether direct induction of redox stress by treatment with H_2_O_2_ would result in comparable autophagy modulation as PBCA NPs. Indeed, when RPE-1 cells were treated with H_2_O_2_ at increasing concentrations for 1 h, Torin1-induced LC3 flux was inhibited (Supplementary Figure S13). In line, H_2_O_2_ inhibited Torin1-induced mKeima-LC3 processing in a concentration-dependent manner, which was fully reversed by NAC (Figure 7a). Treatment with H_2_O_2_ also inhibited Torin1-induced LDHB-mKeima processing, and this was fully abolished by NAC (Figure 7b,c).

On the other hand, H_2_O_2_ was not able to mimic the autophagy-potentiating effect observed at low concentrations of PBCA, not even when cells were treated with lower concentrations of H_2_O_2_ than those shown in Figure 7 (data not shown). Thus, taken together, redox perturbation seems to be both necessary and sufficient for autophagy inhibition by high PBCA concentrations, whereas it is required, but may not be sufficient for, potentiation of autophagy by low concentrations of PBCA.

### 3.5. PBCA-Induced Redox Imbalance Induces Phosphorylation of Beclin-1 and Bcl-2 via p38 and JNK

Intrigued by the autophagy-potentiating effect of low concentrations of PBCA, we aimed to elucidate the underlying mechanism. The cellular energy sensor AMP-activated protein kinase (AMPK) is known to promote autophagy by inhibiting the mTORC1 pathway and/or by directly phosphorylating ULK1 [40]. Knowing that PBCA depends on the ULK kinases for potentiation of Torin1-induced autophagic degradation, we assessed AMPK phosphorylation and downstream signaling upon treatment with autophagy-potentiating concentrations of PBCA (6.25 µg/mL). To our surprise, PBCA did not activate, but rather seemed to inhibit AMPK phosphorylation, as well as phosphorylation of its downstream substrate acetyl-CoA carboxylase (Supplementary Figure S14). Consequently, AMPK activation does not seem to be responsible for PBCA-induced autophagy potentiation.

**Figure 8:**
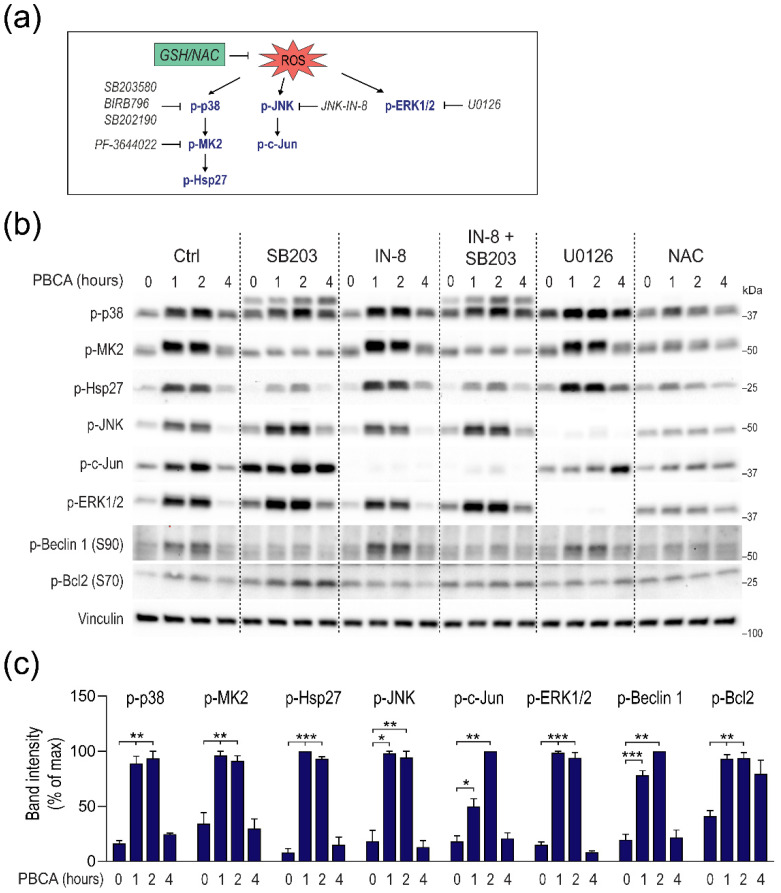
PBCA activates all three major MAPK classes downstream of redox stress. (**a**) Schematic overview of the MAPK pathways with relevant treatments and inhibitors used in this study. (**b**,**c**) RPE-1 cells were pre-treated with SB203580 (SB203, 3 µM), JNK-IN-8 (IN-8, 3 µM), U0126 (10 µM) and NAC (3 mM), as detailed in Materials and Methods, before PBCA (6.25 µg/mL) was added and the incubation continued for the indicated time. Cell lysates were prepared for immunoblotting and the blots were probed with the indicated antibodies. The relative signal for each target was quantified and normalized to the maximum value within each blot (**c**). The graph shows mean values ± SEM quantified from at least three independent experiments. The asterisks denote the statistical significances compared to untreated control. *, *p* < 0.05; **, *p* < 0.01; ***, *p* < 0.001. The dashed lines are introduced in the immunoblot for visual purposes only (i.e., the blot was not cut).

Another set of kinases that are implicated in redox homeostasis and autophagy regulation is the mitogen-activated protein kinases (MAPKs), including the growth factor-related extracellular signal-related kinases (ERKs), and the stress-activated MAPKs, c-Jun N-terminal kinase (JNK) and p38 [41,42,43,44,45,46]. In order to determine whether any of these kinases are involved in PBCA-induced activation of autophagy, we first determined whether kinase activity was altered in cells treated with autophagy-potentiating concentrations of PBCA. Indeed, a 1 h treatment with 6.25 µg/mL PBCA significantly increased phosphorylation of ERK1/2, p38 and JNK themselves, and also of the p38 downstream targets MK2 and Hsp27, and the JNK downstream target c-Jun (Figure 8). The phosphorylation signal remained constant after 2 h of PBCA treatment and returned to basal levels after 4 h. The specificity of the signals was verified by a panel of inhibitors. Indeed, PBCA-induced phosphorylation of MK2 and Hsp27 was specifically abolished by the p38 inhibitors SB203580 and SB202190, or the structurally unrelated p38 inhibitor BIRB 796, whereas PBCA-induced c-Jun phosphorylation was specifically blocked by the JNK1/2/3 inhibitor JNK-IN-8, and PBCA-induced ERK1/2 phosphorylation was abolished by the selective MEK1/2 inhibitor U0126 (Figure 8; Supplementary Figure S15). Importantly, pre-treatment with NAC or GSH strongly diminished PBCA-mediated activation of all the MAPKs (Figure 8b, Supplementary Figure S15), indicating that low concentrations of PBCA activate all three major MAPK classes downstream of redox stress.

Two of the best characterized implications of MAPKs in activation of autophagy are the JNK-mediated phosphorylation of Bcl-2 at residues Thr69, Ser70 and Ser87 that dissociates the Beclin-1-Bcl-2 complex, allowing Beclin-1 to engage in autophagy [42,47,48], and the p38-mediated phosphorylation of the Beclin-1 Ser90 residue, which has been reported to activate autophagy in response to ROS generation or to other stress stimuli [46,49,50]. Consequently, we assessed the phosphorylation status of Beclin-1 and Bcl-2 in PBCA-treated cells. Interestingly, treatment with 6.25 µg/mL PBCA for 1 h significantly enhanced the levels of Beclin-1 phosphorylated at Ser90 and Bcl-2 phosphorylated at Ser70 (Figure 8), and these effects were specifically abolished by co-treatment with p38 inhibitors or JNK-IN-8, respectively (Figure 8b, Supplementary Figure S15). Inhibiting the ERK pathway had no clear effect on phosphorylation of Beclin-1 and Bcl-2. In contrast, NAC or GSH pre-treatment completely eliminated PBCA-induced Beclin-1 and Bcl-2 phosphorylation (Figure 8b, Supplementary Figure S15). Taken together, our results indicate that PBCA-induced redox imbalance induces phosphorylation of the two well-known autophagy-promoting targets, Beclin-1 and Bcl-2 via p38 and JNK.

The biological outcome of MAPK activation is reported to depend on the signal strength and duration. As some reports demonstrate autophagy inhibition downstream of MAPK activation [41,51], we were curious to see how 25 µg/mL PBCA, which inhibits autophagy, would affect MAPK signaling. Treatment of RPE-1 cells with 25 µg/mL PBCA led to persistent activation of p38, JNK and ERK in a ROS-dependent manner, with downstream phosphorylation of both Beclin-1 and Bcl-2 (Supplementary Figure S16A). Nonetheless, the persistent MAPK activation did not seem to be responsible for the PBCA-induced block of autophagy, as co-treatment with MAPK inhibitors did not prevent the blockage of LDHB-mKeima processing (Supplementary Figure S16B).

### 3.6. PBCA Activates Autophagy Downstream of p38 and JNK Activation

**Figure 9:**
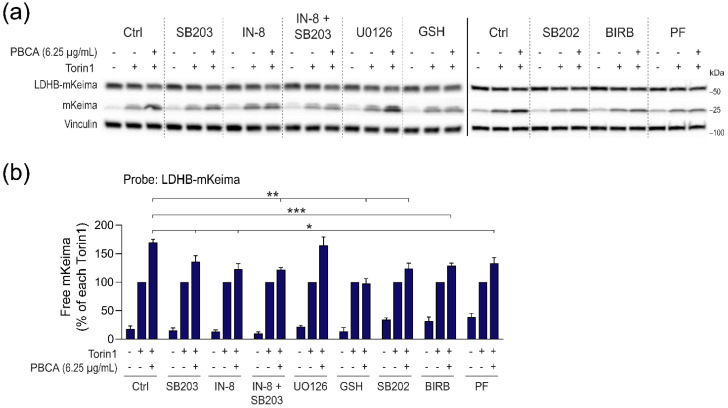
PBCA-mediated potentiation of Torin1-induced autophagy is dependent on the p38–MK2 pathway and JNK1/2. RPE-1 cells with inducible expression of LDHB-mKeima were induced for 2 d, washed, and pre-treated with SB203580 (SB203, 3 µM), JNK-IN-8 (IN-8, 3 µM), U0126 (10 µM), GSH (10 mM), SB202190 (SB202, 3 µM), BIRB 796 (BIRB, 10 µM), or PF-3644022 (PF, 2.5 µM) before addition of Torin1 (50 nM) and PBCA (6.25 µg/mL). The incubation was continued for 4 h before cell lysates were prepared for immunoblotting (**a**), and the relative amounts of free mKeima were quantified (**b**). The data were normalized to the respective Torin1-treated sample for each inhibitor. The graph shows mean values ± SEM quantified from at least three independent experiments. The asterisks denote the statistical significances compared to Torin1 + PBCA alone. *, *p* < 0.05; **, *p* < 0.01; ***, *p* < 0.001. Solid line demarks samples from two different gels, whereas the dashed lines are introduced for visual purposes only (i.e., the membranes were not cut).

Since 6.25 µg/mL of PBCA enhanced the phosphorylation of Beclin-1 and Bcl-2 downstream of MAPKs, we were eager to learn whether MAPK activation is essential for PBCA-induced potentiation of autophagy. ERK activity did not seem to be involved, as treatment with the MEK inhibitor U0126 did not reduce PBCA-mediated potentiation of autophagy (Figure 9).

In contrast, inhibition of JNK with JNK-IN-8, or inhibition of p38 with any of the three above-mentioned p38 inhibitors significantly reduced PBCA-mediated enhancement of LDHB-mKeima processing (Figure 9). Moreover, the stimulatory effect of PBCA on Beclin-1 phosphorylation and LDHB-mKeima processing was reduced by the MK2 inhibitor PF-3644022 (Figure 9, Supplementary Figure S15), indicating that p38 signaling via MK2 is required for Beclin-1 Ser90 phosphorylation and the subsequent stimulation of autophagy. Interestingly, combined treatment with JNK-IN-8 and SB203580 did not further reduce LDHB-mKeima processing (Figure 9), suggesting that JNK and p38 converge on regulating the same downstream pro-autophagic mechanism.

**Figure 10:**
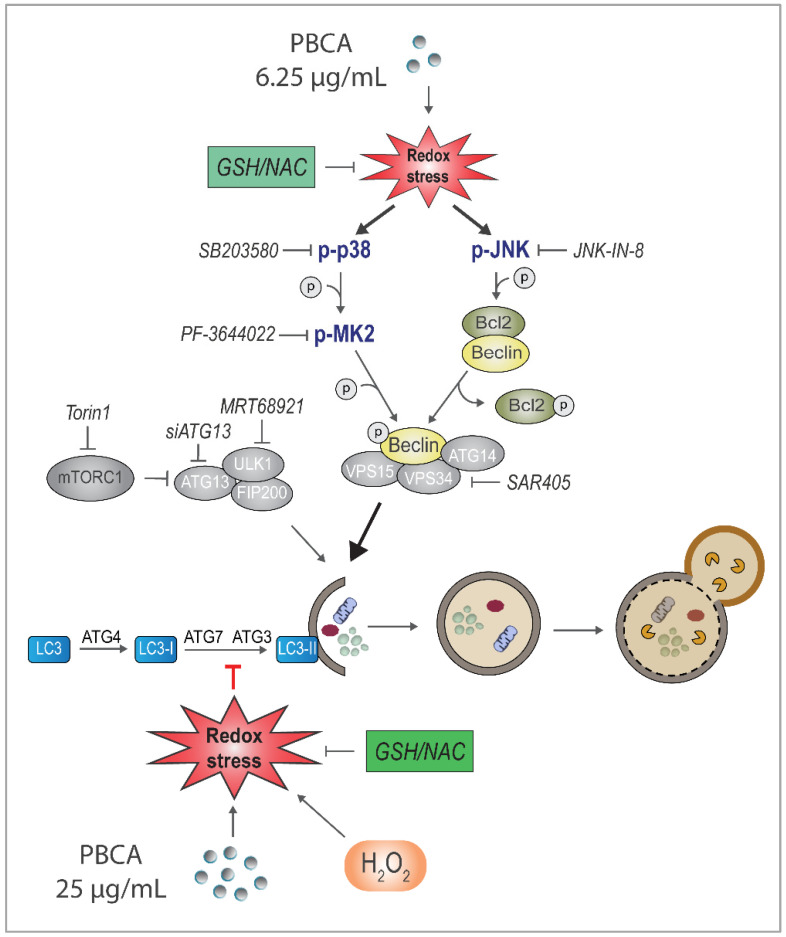
Model of PBCA-induced cellular effects, highlighting our most important findings. Treatment with PBCA promotes functional autophagy at low concentrations and inhibits autophagy at high concentrations depending on the level of redox homeostasis perturbation. Both effects were completely abolished by the antioxidants NAC and GSH. High concentrations of PBCA inhibited MAP1LC3B/GABARAP lipidation and blocked autophagic carrier and cargo flux induced by Torin1. The effects were mimicked by the redox regulator H_2_O_2_. Low concentrations of PBCA enhanced autophagic cargo flux and degradation in an ULK1/2-, ATG13- and Vps34-dependent manner, yet without a concomitant increase in LC3 lipidation or flux. PBCA activated MAP kinase signaling cascades in a redox-dependent manner, and interference with individual signaling components revealed that the autophagy-stimulating effect of PBCA required the action of the JNK and p38–MK2 pathways, whose activities converged on the pro-autophagic protein Beclin-1 (“Beclin”).

Of note, PBCA-induced MAPK activation was unaffected by Torin1, and NAC prevented the activation under both nutrient-rich, basal conditions and Torin1-induced conditions (Supplementary Figure S17). Finally, in line with the data obtained with NAC (Figure 6f), supplementing the medium with GSH completely abolished PBCA-induced potentiation of LDHB-mKeima processing (Figure 9), supporting all indications of redox stress being a major player in autophagy regulation by PBCA. In summary, Figure 10 depicts a simplified model of PBCA-mediated regulation of autophagy, based on our results.

## 4. Discussion

In this work we demonstrated that oxidative stress induced by NP treatment is a factor that is crucial in determining its regulation of autophagy. By using the well-known poly(alkyl cyanoacrylate) NP PBCA we showed how the magnitude of NP-induced redox imbalance correlates with the modulation of autophagy from a stimulating effect to an inhibitory effect. Importantly, these effects were unrelated to any NP-induced cytotoxicity. At low concentrations, PBCA-induced redox imbalance was required to increase bulk autophagy flux and cargo degradation via activation of p38–MK2 and JNK pathways, which likely converge on enhancing the pro-autophagic function of Beclin-1. PBCA promoted a canonical form of autophagy that, intriguingly, proceeded without a concomitant increase in LC3 flux. At high concentrations, the more severe PBCA-induced redox imbalance caused a strong inhibition of both LC3 lipidation and bulk autophagic cargo flux, which was not mediated by MAPK activation and which could be mimicked by direct redox stress induction with H_2_O_2._

It is important to understand how NPs influence autophagy, since alterations in autophagic activity may have profound impacts on cells and organisms, and can affect patient responses in therapeutic settings [7,8,9]. The autophagy and nanotechnology fields, including the field of nanomedicine, have experienced an exponential growth in the last 15 years, and with it several hundreds of publications on the effect of NPs on autophagy have been produced. This has resulted in much insight into potential autophagy-regulatory mechanisms induced by NPs. However, autophagy is a highly dynamic and complex process, which requires very thorough analysis [23], and the progress in understanding how NPs regulate autophagic activity is currently hampered by a general lack of using functional methods that monitor autophagic *cargo* flux instead of relying predominantly, or only, on the use of LC3 or other autophagic markers. To the best of our knowledge, the current work represents the first to use a whole panel of functional autophagy assays to explore NP-mediated effects on autophagy.

This thorough approach enabled us to identify that low concentrations of PBCA (6.25 μg/mL) potentiate autophagy in human epithelial cells. Remarkably, PBCA enhanced autophagic cargo flux and degradation without inducing any alterations in the levels of lipidated LC3 (LC3-II), nor in the flux of LC3 through the autophagic pathway. Thus, if we had based our approach only on LC3-based assays, we would have completely missed the pro-autophagic effect of PBCA. This strongly highlights the need to employ a functional, LC3-independent assay to explore the effect of NPs (or of any other treatment or condition) on autophagy. Our findings suggest that low concentrations of PBCA trigger a form of autophagy that is independent of LC3-associated autophagosomal membranes, and which likely is independent of LC3 altogether. Nevertheless, PBCA-induced autophagy appeared to be canonical, since it was dependent on the key canonical autophagy machinery components Vps34, ULK1/2 and ATG13. LC3-independent canonical autophagy has previously been described in conditions of amino acid starvation [52] or ER stress [53] in prostate cancer cells, and very recently also in lung cancer cells treated with ALK inhibitors [54]. Moreover, in amino acid-starved and cycloheximide-treated primary rat hepatocytes, LC3 flux ceases within 2 h, whereafter proficient flux of endogenous autophagic cargo goes on uninterruptedly even in the complete absence of LC3 flux [52]. Together, those and our current results indicate that the phenomenon of LC3 flux-independent autophagy can occur under diverse conditions and in different cell types, and is thus worthy of more attention. Since redox imbalance appears to be involved in autophagy induced by both amino acid starvation [20,55] and PBCA (current study), it will be interesting to see if LC3 flux-independent autophagy is specifically related to redox stress, or whether it is an even broader phenomenon.

We demonstrated that low concentrations of PBCA activates p38 and JNK MAPKs in a redox-dependent manner, and that both p38 and JNK were largely required for PBCA-mediated autophagy stimulation. Furthermore, we showed that PBCA-induced p38 activation mediates an increase in phosphorylation of Beclin-1 at the Ser90 residue. Beclin-1 phosphorylation was first observed upon starvation-induced autophagy, and the two phosphorylation sites Ser90 and Ser93 were identified as contributing to the autophagic function of Beclin-1 [49]. Phosphorylation of Beclin-1 at these residues was later established to depend on the downstream kinases of p38, MK2 and MK3, and the phosphorylated Beclin-1 contributed to increase the lipid kinase activity of the Vps34 complex [46]. Other kinases have also been implicated in phosphorylation of Beclin-1 Ser90, including DAPK3 [50], CaMKII [56], and AMPK [57]. Since the MK2 inhibitor PF-3644022 strongly reduced both Beclin-1 phosphorylation and PBCA-stimulated autophagy, it is likely that MK2 is the major contributor to Beclin-1 Ser90 phosphorylation in PBCA-treated cells. Nevertheless, we cannot formally exclude that the elevated levels of p-Beclin-1 Ser90 may be caused by decreased dephosphorylation activity. Such a phenomenon was observed in MCF7 breast cancer cells treated with okadaic acid, which inhibits the phosphatase PP2A implicated in Beclin-1 dephosphorylation [50]. Autophagy activation through Beclin-1 Ser90 phosphorylation has been shown to require upstream JNK1 activity [46], and thus to be a collaborative action of the p38–MK2 and JNK signaling pathways. JNK-mediated phosphorylation of Bcl-2 dissociates the Beclin-1-Bcl-2 complex, thereby removing the steric blockade imposed by Bcl-2 [46]. Our data are in line with such a collaborative action of p38 and JNK towards Beclin-1 in PBCA-mediated potentiation of autophagy at low NP concentrations. The potentiation was strongly inhibited when either kinase was blocked separately, and concomitant inhibition of both p38 and JNK did not result in a further inhibition of PBCA-induced autophagy, thus indicating convergence of the pathways.

Although PBCA-induced potentiation of autophagy was fully abolished upon co-treatment with an excess of antioxidants, the autophagy-promoting effects of PBCA could not be mimicked by H_2_O_2_. This suggests that the PBCA-provoked redox imbalance is necessary, but not sufficient, for autophagy induction. However, the possibility exists that PBCA treatment may induce other types of ROS than H_2_O_2_, such as mitochondrial ROS or lipid ROS, and that such ROS-production is mediating PBCA-induced autophagy, and not that produced from H_2_O_2_. Alternatively, autophagy potentiation may not be mediated by increased ROS production per se, but rather by decreased GSH levels. Activation of starvation-induced autophagy is for instance associated with reduced GSH levels [55]. This would be in line with the rapid reduction of GSH and the more delayed accumulation of ROS that we observed upon treatment with PBCA. Similar to the autophagy-promoting effects of low PBCA concentrations, the autophagy-inhibitory effects observed at high PBCA concentrations were abolished upon co-treatment with an excess of antioxidants. However, in contrast to the autophagy-promoting effects, the autophagy-inhibitory effects of PBCA were fully mimicked by H_2_O_2_. This indicates that the redox imbalance provoked by high PBCA concentrations is both required and sufficient for its autophagy-inhibitory effects. Taken together, our data obtained with PBCA NPs suggest a shift in redox imbalance severity with increasing NP concentrations, and perhaps also in the sources and intracellular localization of the increased ROS production, and that this dictates the NP-effect on autophagy.

Alterations in redox balance are frequently reported to be the underlying mechanism of NP-induced cellular effects [10,11,12]. However, with the exception of metal-based NPs that may directly generate ROS through Fenton and Fenton-like reactions [58,59], it is completely unknown how NPs alter cellular redox capacity. The nano–bio interface includes interactions between NPs and proteins, cellular membranes and biofluids, all of which may directly or indirectly influence cellular redox states [12]. Thus, the initiating mechanisms may be very diverse, and their deciphering will require careful and encompassing studies.

Interestingly, the entry point/molecular targets in the autophagic pathway appear to differ with respect to the autophagy-promoting versus autophagy-inhibitory effects of PBCA. Thus, whereas LC3-II levels were unaffected by low PBCA concentrations, high PBCA concentrations strongly reduced the levels of LC3-II. The latter is most likely due to inhibition of LC3 lipidation, since LC3 flux and processing (which has the potential of reducing LC3-II levels) were blocked by PBCA. To the best of our knowledge, PBCA is the first NP shown to reduce LC3-II protein levels due to reduced lipidation, and, notably, we have observed this reduction under both nutrient-rich, basal conditions [27] and when autophagy is activated (this study). The rapid loss in the ability to form LC3-II suggests that the effect is not due to transcriptional regulation of LC3 levels, but rather post-translational modifications of autophagy regulators. Since pretreatment with NAC or GSH completely prevented LC3 inhibition, and the inhibitory phenotype was mimicked by treatment with H_2_O_2_, our data clearly demonstrate that PBCA-mediated inhibition of LC3 lipidation is downstream of redox imbalance induced by the NP treatment. Interestingly, such a regulatory mechanism would be in line with a recently described molecular mechanism underlying impaired autophagy due to direct oxidation of ATG3 and ATG7 [19], the E1- and E2-like enzymes that mediate the conjugation of LC3 to PE on the forming autophagosome [60]. It was reported that ATG3 and ATG7 are more prone to thiol oxidation (redox regulation) during activation of autophagy since the stable thioester interaction with LC3 is lost. This allows for the formation of intermolecular disulfide-bound complexes incapable of transferring LC3 to phosphatidylethanolamine during active autophagy [19]. This may explain why the PBCA-induced inhibition of LC3 lipidation is far stronger when autophagy is activated than under basal conditions. Moreover, this regulatory mechanism implicates alterations in GSH levels, which is in accordance with the rapid decrease in GSH levels upon PBCA treatment. Rapid depletion of GSH may be caused by either GSH efflux [55], or consumption of GSH through formation of oxidized glutathione during ROS detoxification [61]. Increased levels of oxidized glutathione were shown to inhibit the enzymatic activity of the catalytic thiols on ATG3 and ATG7 by S-glutathiolation in a cell-free reaction [19]. Thus, it is tempting to speculate that PBCA-mediated depletion of GSH inhibits LC3 lipidation through redox regulation of ATG3 and ATG7. Curiously, ATG4 has been shown to be subjected to a comparable redox regulation, but the proposed outcome on autophagy is different [20]. During starvation-induced autophagy, the ATG4 protease was reported to be a direct target for oxidation by H_2_O_2_, leading to inactivation of ATG4 only at the site of autophagosome formation, which would selectively inhibit the de-conjugation step of ATG8 (LC3/GABARAPs), thus promoting lipidation [20]. Based on this discrepancy, it is tempting to speculate that redox stress as autophagy modulator depends on multiple factors, including timing, localization, and type of oxidative imbalance or severity of the oxidative stress. Future deciphering of such associations will be essential to enwiden our understanding of how NPs and redox imbalances regulate autophagy in human cells.

## 5. Conclusions

PBCA-induced redox imbalance regulates autophagy in a concentration-dependent manner. At low concentrations of PBCA, the cells are able to increase autophagic degradation through phosphorylation of Beclin-1 and Bcl-2 by p38–MK2 and JNK MAPK pathways, respectively, most likely as an attempt to regain cellular homeostasis. At high concentrations, autophagy is rapidly inhibited, and cell viability is eventually compromised. Our findings may have important implications for the therapeutic use of PBCA NPs, as well as for aspects related to other NPs and other conditions that induce cellular redox imbalance.

## Data Availability

Not applicable.

## Supplementary Materials

The following are available online at https://www.mdpi.com/article/10.3390/cells10123432/s1: Figure S1: Determination of the autophagic fraction of long-lived protein degradation (LLPD) through use of the specific autophagy inhibitor SAR405, Figure S2: The lactate dehydrogenase (LDH) sequestration assay, Figure S3: The rapid autophagy-inhibiting effect of PBCA at 25 µg/ml is not caused by loss of cell viability or induction of apoptosis, Figure S4: RPE-1 cells with inducible expression of LDHB-mKeima, Figure S5: PBCA stimulates autophagic cargo flux at low NP concentrations and inhibits autophagy at high concentrations, Figure S6: Verification of the functionality of RPE-1 cells with inducible expression of mKeima-LC3, Figure S7: Treatment with PBCA persistently inhibits LC3 flux and inhibits GABARAP flux at 1 h, Figure S8: The PBCA-mediated potentiation of Torin1-induced autophagy is abolished by SAR405, inhibition of ULK1/2 activity, or depletion of ATG13, Figure S9: PBCA-mediated stimulation of autophagy is mTORC1-independent, Figure S10: Treatment with PBCA leads to generation of ROS, Figure S11: NAC prevents PBCA-mediated inhibition of Torin1-induced mKeima-LC3 degradation, Figure S12: Treatment with NAC or GSH reduces the cytotoxicity induced by long-term PBCA incubation, Figure S13: Treatment with H_2_O_2_ reduces Torin1-induced LC3 flux, Figure S14: Treatment with PBCA inactivates AMPK phosphorylation and downstream signalling, Figure S15: Verification of the specificity of the p38α/β inhibitor SB202190, the pan-p38 inhibitor BIRB 796, and the MK2 inhibitor PF-3644022, Figure S16: High concentration of PBCA induces ROS-dependent, prolonged activation of signalling downstream of p38 and JNK, but this signalling is not responsible for PBCA-mediated inhibition of LDHB-mKeima processing, Figure S17: PBCA-mediated activation of signalling downstream of p38 and JNK is unaltered in the presence of Torin1, and abolished by NAC.
