# Supplementary material for: Perturbation of Cellular Redox Homeostasis Dictates Divergent Effects of Polybutyl Cyanoacrylate (PBCA) Nanoparticles on Autophagy
Source: Cells. 2021 Dec 6;10(12):3432. doi: 10.3390/cells10123432 (PMC8699722; doi:10.3390/cells10123432)
Supplement: Supplementary file 1 [file cells-10-03432-s001.zip › Supplementary Figures.pdf]

Supplementary figures

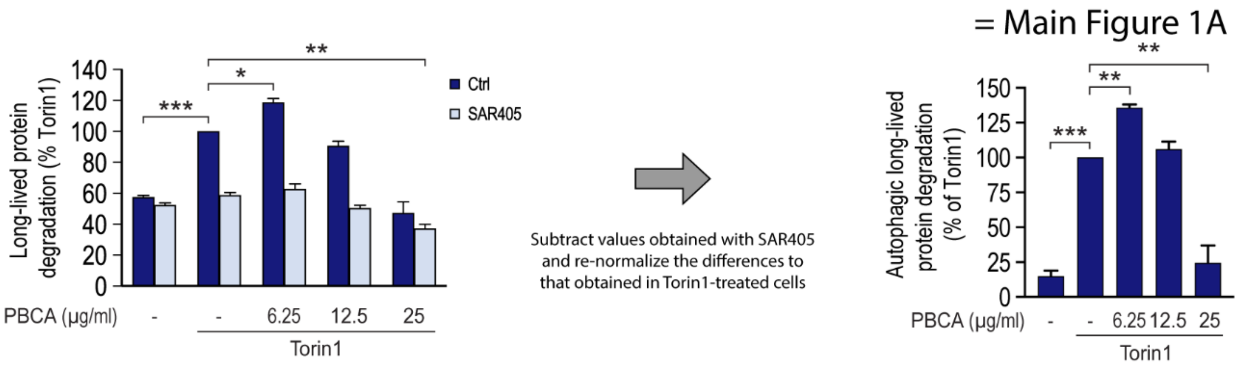

Supplementary Figure S1. Determination of the autophagic fraction of long-lived protein degradation (LLPD) through use of the specific autophagy inhibitor SAR405. RPE-1 cells were radiolabeled with [^{14}C]valine for 1 d and chased for 3 h. To enable calculation of the autophagic fraction of LLPD, the cells were subsequently treated with Torin1 (50 nM) and the indicated concentrations of PBCA in the absence or presence of SAR405 (10 µM) for 4 h, as described in Materials and Methods. Left panel: The LLPD raw data, normalized to Torin1 alone. Right panel: The difference in LLPD between Ctrl and SAR405-treated cells for each condition, i.e. the autophagic fraction of LLPD, re-normalized to Torin1. The right panel is identical to main Figure 1a. *, $p < 0.05$; **, $p < 0.01$; ***, $p < 0.001$.

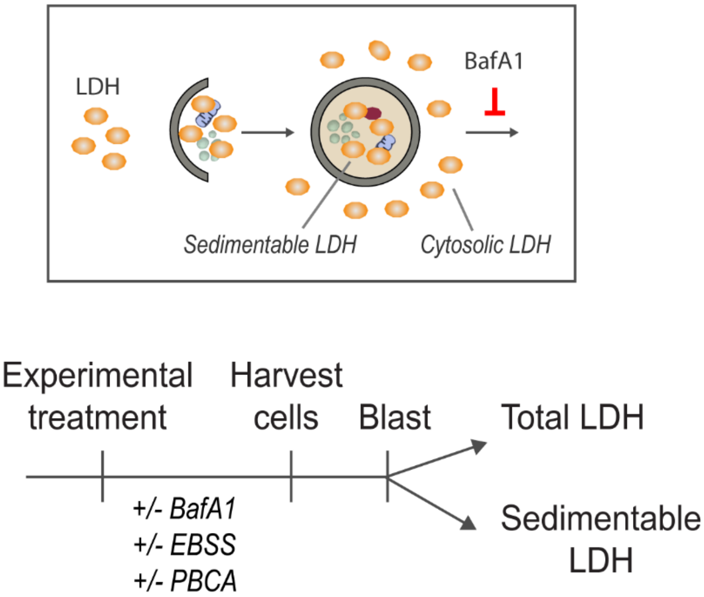

Supplementary Figure S2. The lactate dehydrogenase (LDH) sequestration assay. The LDH sequestration assay is a method to quantitatively measure the sequestration rate of bulk cytosolic cargo into closed autophagosomes by using the endogenous cytosolic protein LDH as a marker. BafA1 was used as an inhibitor of lysosomal degradation to quantify the net sequestration rate of LDH independent of its degradation. In our experimental setup, the cells were subjected to EBSS/FBS amino acid-free medium as an inducer of autophagy, in the absence or presence of PBCA. Cells were harvested, and the plasma membrane disrupted by a short electric pulse leaving the membranes of organelles and vesicles intact. As such, *Sedimentable LDH*, can be separated from cytosolic LDH by centrifugation, and the amount of *Sedimentable LDH* to *Total LDH* measured by enzymatic activity. LDH sequestration activity is calculated by subtracting the background of sedimentable LDH in non-treated control cells.

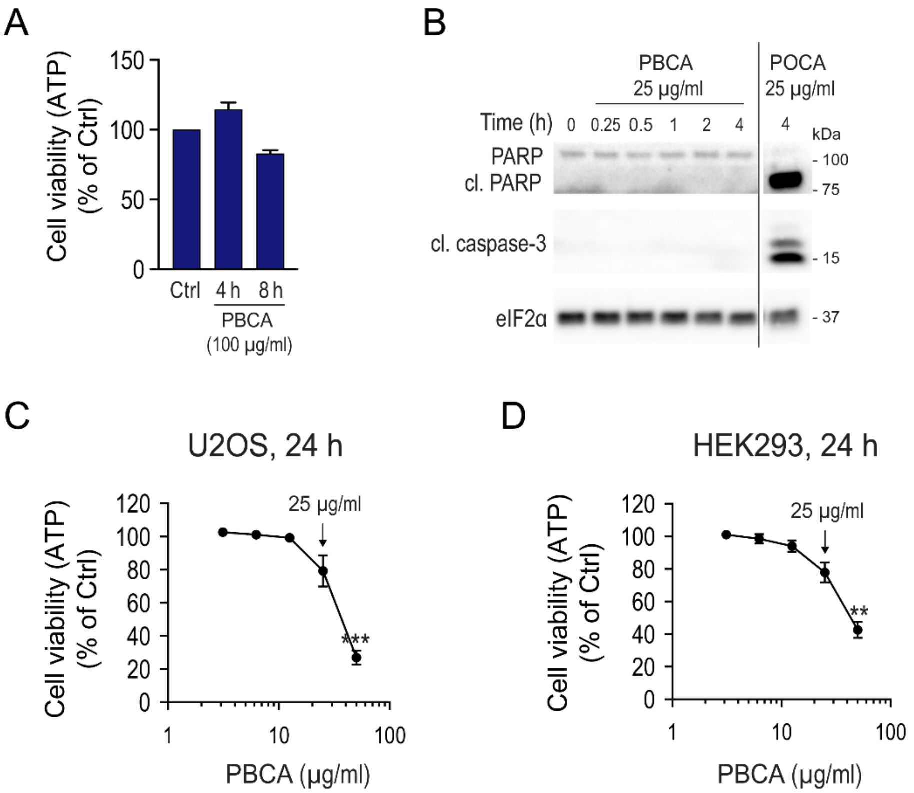

Supplementary Figure S3. The rapid autophagy-inhibiting effect of PBCA at 25 µg/ml is not caused by loss of cell viability or induction of apoptosis. A. Cell viability assessed by ATP levels of RPE-1 cells treated with PBCA (100 µg/ml) for 4 and 8 h from one experiment. The graph shows mean values ± standard deviation of triplicates. **B.** RPE-1 cells were treated with PBCA (25 µg/ml) for the indicated time points. POCA (25 µg/ml, 4 h) was added as a positive control for caspase activation, as demonstrated by Szwed et al. (2019) [2]. Cell lysates were prepared for immunoblotting and the blots were probed with the indicated antibodies. Solid line indicates where the blot was cut for illustration. **C-D.** Cell viability assessed by ATP levels of U2OS cells (C) or HEK293 cells (D) treated with PBCA at increasing concentrations for 24 h. The curves show mean values ± SEM quantified from at least three independent experiments. The asterisks denote the statistical significances compared to untreated control cells **, $p < 0.01$; ***, $p < 0.001$.

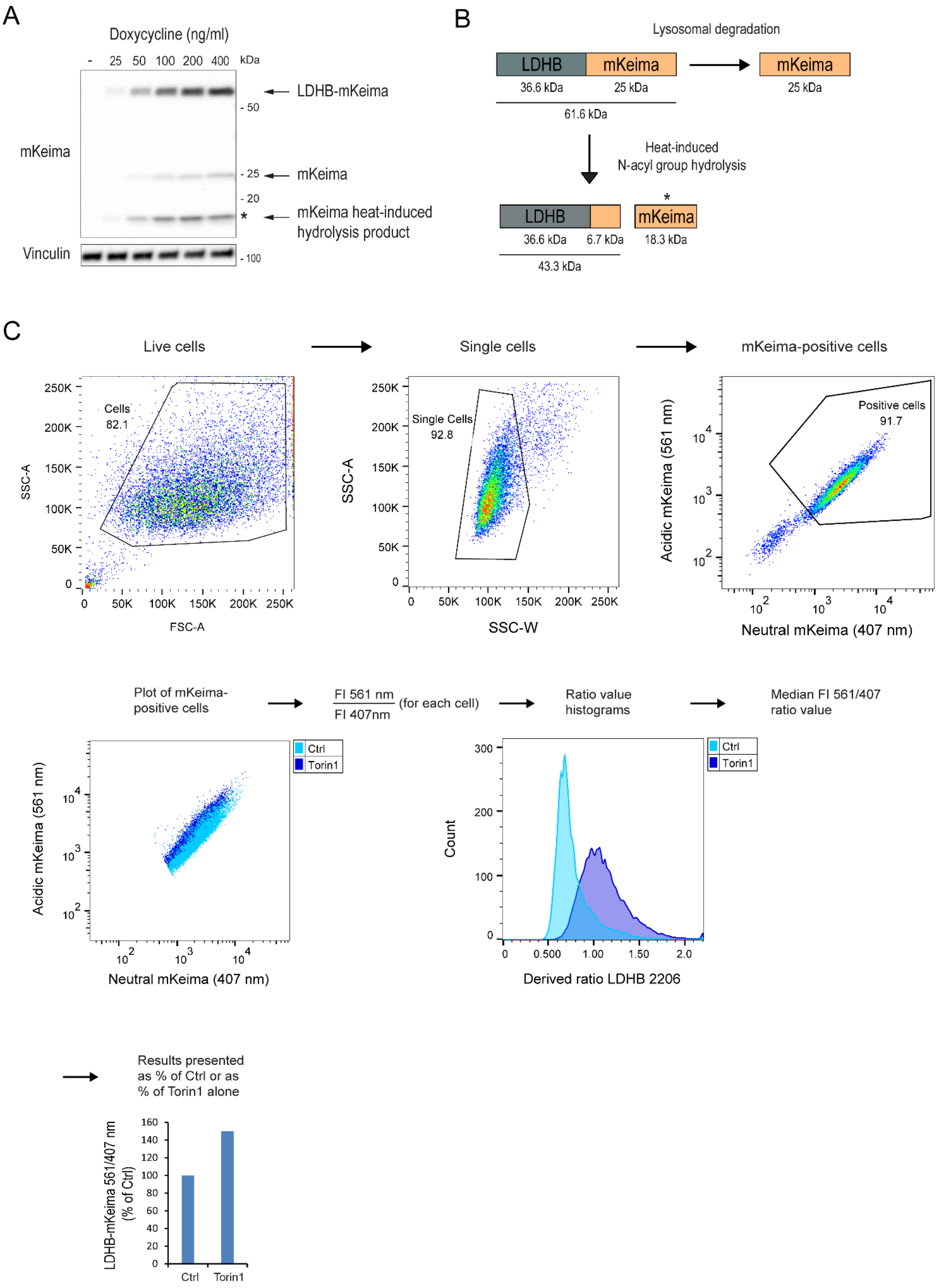

A
Doxycycline (ng/ml)
- 25 50 100 200 400 kDa
mKeima
LDHB-mKeima
- 50
- 25
mKeima
- 20
*
mKeima heat-induced hydrolysis product
Vinculin
- 100
B
Lysosomal degradation
LDHB
mKeima
36.6 kDa
25 kDa
61.6 kDa
mKeima
25 kDa
Heat-induced N-acyl group hydrolysis
LDHB
36.6 kDa
6.7 kDa
43.3 kDa
*
mKeima
18.3 kDa
C
Live cells
Single cells
mKeima-positive cells
Cells 82.1
SSC-A
FSC-A
Single Cells 92.8
SSC-W
Positive cells 91.7
Acidic mKeima (561 nm)
Neutral mKeima (407 nm)
Plot of mKeima-positive cells
FI 561 nm / FI 407nm (for each cell)
Ratio value histograms
Median FI 561/407 ratio value
Ctrl
Torin1
Count
Derived ratio LDHB 2206
Results presented as % of Ctrl or as % of Torin1 alone
LDHB-mKeima 561/407 nm (% of Ctrl)
Ctrl
Torin1

Supplementary Figure S4. RPE-1 cells with inducible expression of LDHB-mKeima. A. Titration of the optimal doxycycline concentration for induction of LDHB-mKeima in RPE-1 cells. Cell lysates were prepared for immunoblotting and the blots were probed with the indicated antibodies. An induction time of 48 h, as shown here, was found to be sufficient. Free mKeima appears as a band migrating at 25 kDa. Additionally, the weak band marked with an asterisk at ~18 kDa represents the C-terminal hydrolysis product generated during boiling of the samples, as previously described by An and Harper (2018) [1]. **B.** Schematic drawing of the two products of LDHB-mKeima obtained by either lysosomal degradation or heat-induced hydrolysis of the N-acyl imine in mKeima. The latter produces a 18.3 kDa C-terminal fragment that is released, and the remaining 6.7 kDa N-terminal fragment of mKeima that is still fused to LDHB appears as a ~40 kDa band at high exposures [1]. **C.** Gating strategy for flow cytometric determination of LDHB-mKeima lysosomal delivery. After 48 h of induction with 100 ng/ml doxycycline, the LDHB-mKeima RPE-1 cells were washed twice to remove doxycycline and were then treated as described in each figure legend. The cells were then detached and subjected to flow cytometry with excitation at 407 and 561 nm and emission at 620 nm for both. The subsequent gating was performed by using the software FlowJo™ (BD Biosciences). First a gate was set around the population of live cells in a FSC-A/SSC-A plot (top left panel). This gating excludes cell debris and dead cells. Correct gating was confirmed by live/dead staining with propidium iodide. Next, single cells were identified by plotting SSC-W versus SSC-A (top middle panel), and the mKeima-positive cells among the live, single cells were identified by plotting the fluorescent signals obtained from excitation with the 407 nm laser versus the 561 nm laser (top right panel). Then the extent of lysosomal delivery of mKeima was determined by ratiometric analyses by using the "Derived Parameters" function in FlowJo. For each cell from each sample the fluorescence intensity obtained with the 561 nm laser was divided by that obtained with the 407 nm laser, and the ratio values were plotted as histograms for each treatment condition (bottom left and middle panel). Finally, the median ratio value from each sample was normalized to that obtained from untreated control cells or to cells treated with Torin1 alone, as specified in each figure.

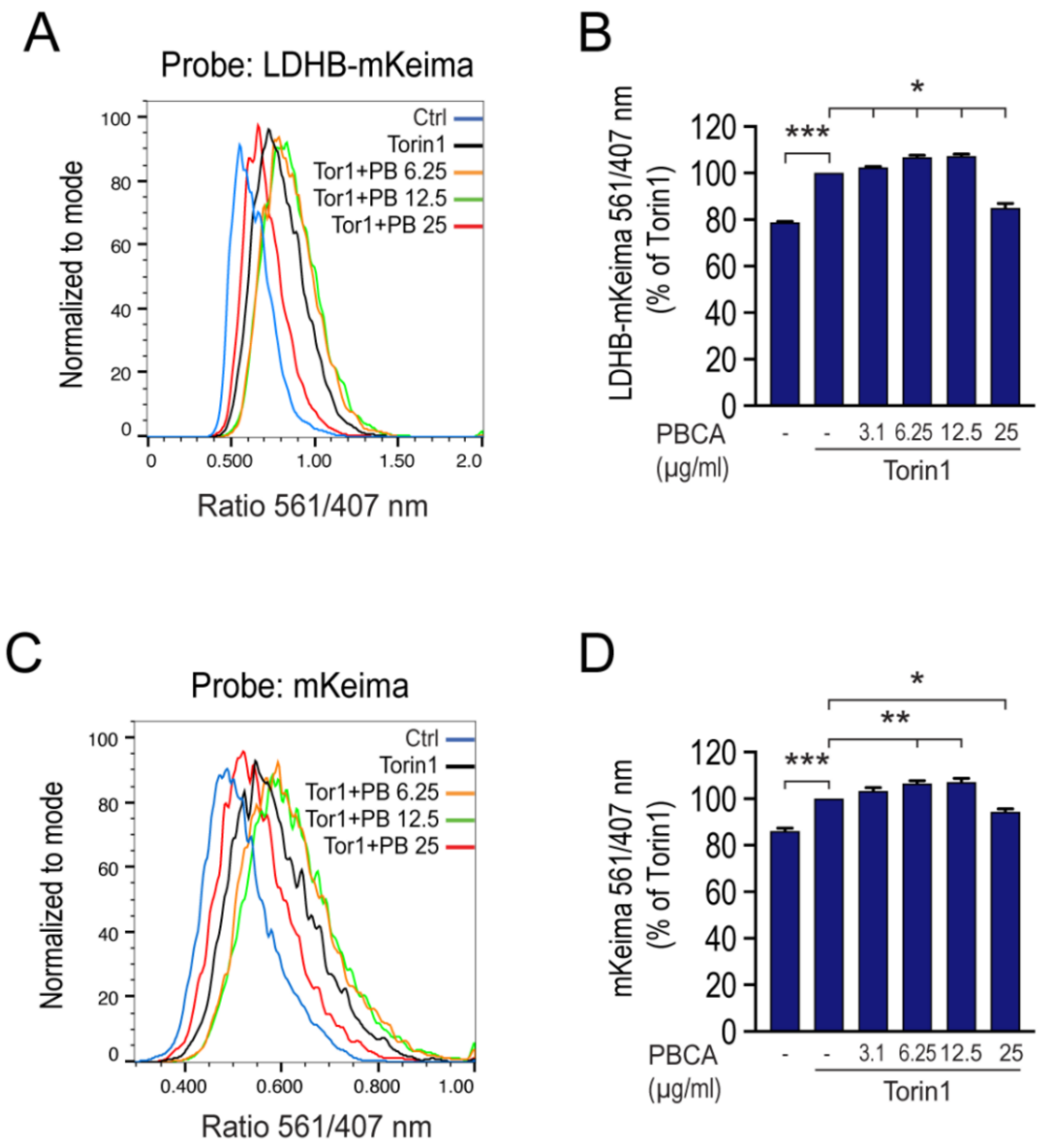

Supplementary Figure S5. PBCA stimulates autophagic cargo flux at low NP concentrations and inhibits autophagy at high concentrations. RPE-1 cells with inducible expression of LDHB-mKeima (**A-B**) or mKeima (**C-D**) were induced for 2 d or 1 d, respectively, washed, and treated with PBCA at the indicated concentrations in the absence or presence of Torin1 (50 nM) for 4 h. The cells were harvested, and the 561/407 nm fluorescence intensity ratios were determined by flow cytometry. Individual ratio histograms from one representative experiment from each cell line are shown (A, C), and the relative median values of the 561/407 nm ratios were quantified (B, D). All graphs show mean values ± SEM quantified from at least three independent experiments. The asterisks denote the statistical significances compared to treatment with Torin1 alone. *, $p < 0.05$; **, $p < 0.01$; ***, $p < 0.001$.

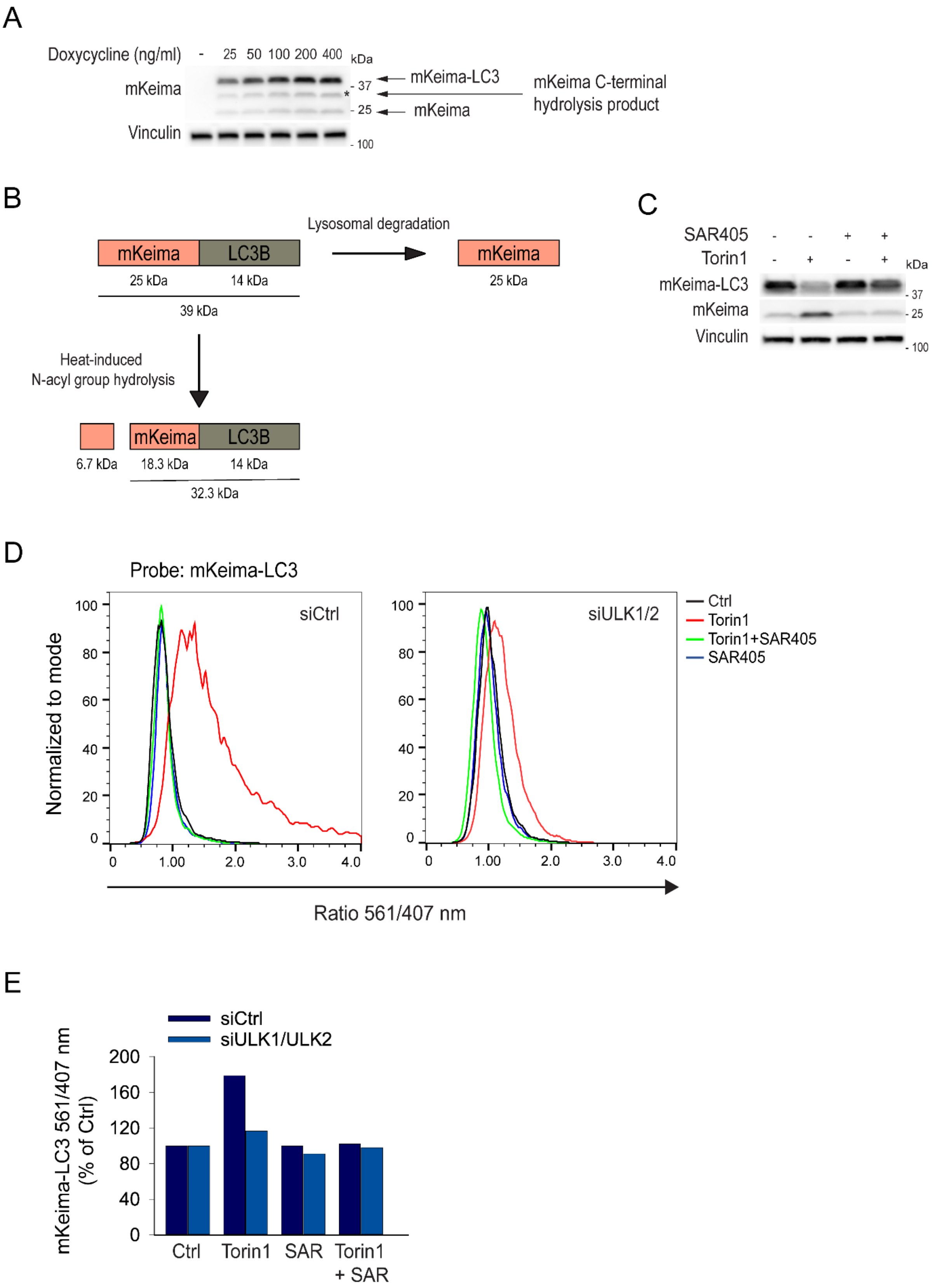
A
Doxycycline (ng/ml)
-
25
50
100
200
400
kDa
mKeima
Vinculin
37
25
100
*
mKeima-LC3
mKeima
mKeima C-terminal
hydrolysis product
B
mKeima
LC3B
25 kDa
14 kDa
39 kDa
Lysosomal degradation
mKeima
25 kDa
Heat-induced
N-acyl group hydrolysis
mKeima
LC3B
6.7 kDa
18.3 kDa
14 kDa
32.3 kDa
C
SAR405
Torin1
kDa
mKeima-LC3
mKeima
Vinculin
37
25
100
D
Probe: mKeima-LC3
siCtrl
siULK1/2
Normalized to mode
Ratio 561/407 nm
Ctrl
Torin1
Torin1+SAR405
SAR405
E
siCtrl
siULK1/ULK2
mKeima-LC3 561/407 nm
(% of Ctrl)
Ctrl
Torin1
SAR
Torin1
+ SAR

Supplementary Figure S6. Verification of the functionality of RPE-1 cells with inducible expression of mKeima-LC3. A. Titration of the optimal doxycycline concentration for induction of mKeima-LC3 in RPE-1 cells. Cell lysates were prepared for immunoblotting and the blots were probed with the indicated antibodies. An induction time of 24 h, as shown here, was found to be sufficient. Free mKeima appears as a band migrating at 25 kDa. Additionally, the weak band marked with an asterisk at 32 kDa represents the C-terminal hydrolysis product generated during boiling of the samples, as previously described by An and Harper (2018) [1]. **B.** Schematic drawing of the two products of mKeima-LC3 obtained by either lysosomal degradation or heat-induced hydrolysis of the N-acyl imine in mKeima. The latter produces a 6.7 kDa N-terminal fragment that is released, and the remaining 18.3 kDa C-terminal fragment of mKeima that is still fused to LC3 appears as the 32 kDa band [1]. **C.** After 24 h of induction with 100 ng/ml doxycycline, the mKeima-LC3 RPE-1 cells were washed twice to remove doxycycline and were then treated with Torin1 (50 nM) for 4 h in the absence or presence of SAR405 (10 µM) to demonstrate that the 25 kDa lysosomal cleavage fragment of mKeima-LC3 only appears upon activation of autophagy. **D-E.** mKeima-LC3 RPE-1 cells were transfected with either non-targeting control siRNA (siCtrl) or two siRNAs targeting ULK1 and ULK2, respectively (siULK1/2). After 24 h the cells were treated with doxycycline to induce expression of mKeima-LC3 and 24 h later the cells were washed twice and treated with Torin1 (50 nM) and SAR405 (10 µM) for 4 h. The cells were detached and subjected to flow cytometry with excitation at 407 and 561 nm and emission at 620 nm for both. The histograms of the 561/407 nm ratio for the cell population in each sample are shown in D, whereas the median values of the mKeima ratio normalized to untreated siCtrl samples are shown in E. Induction of autophagy by Torin1 led to a right-shift of the mKeima ratio histogram giving a clear increase in the median ratio value. Depletion of ULK1 and -2 abolished the Torin1-induced shift in mKeima ratio and strongly reduced the median mKeima ratio value.

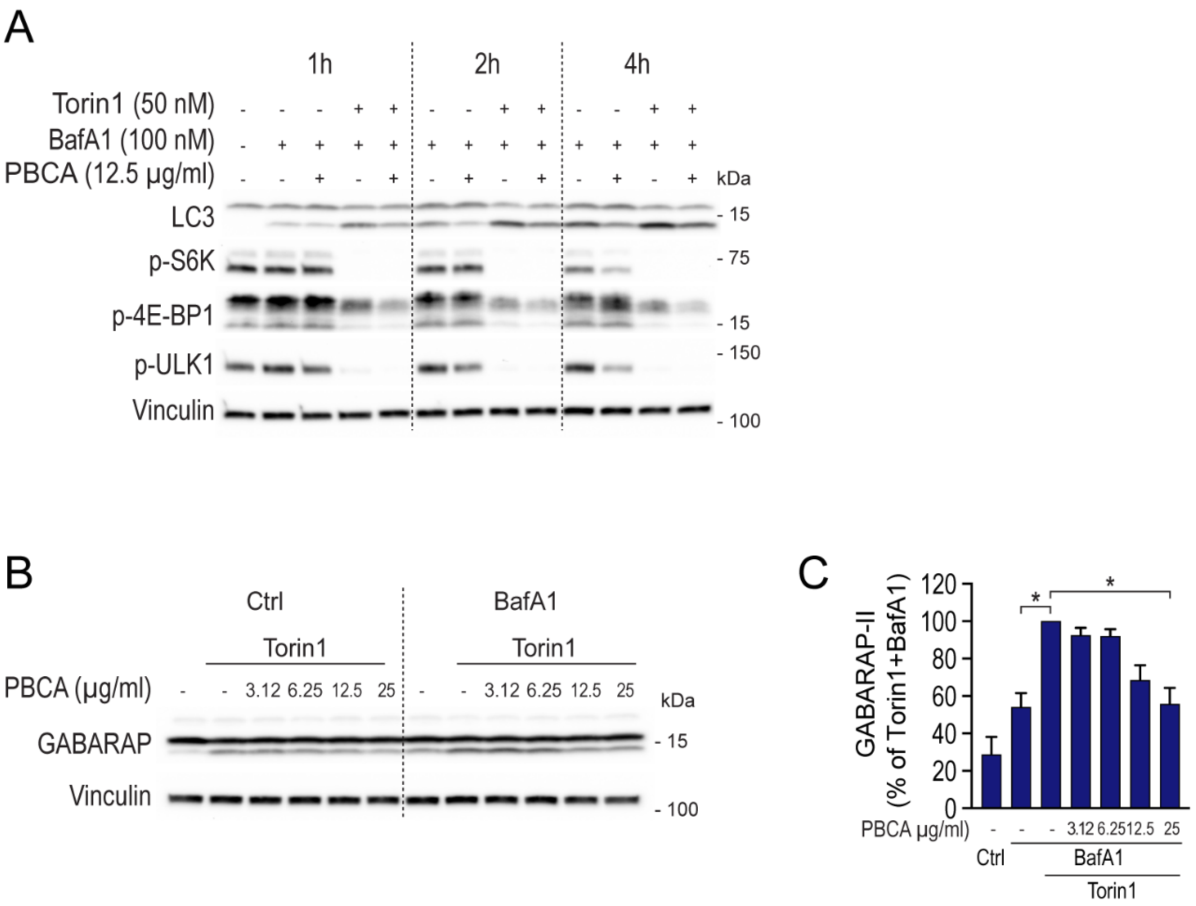

Supplementary Figure S7. Treatment with PBCA persistently inhibits LC3 flux and inhibits GABARAP flux at 1 h. A. RPE-1 cells were treated with PBCA (12.5 µg/ml), Torin1 (50 nM) and BafA1 (100 nM) as indicated for 1 h, 2 h and 4 h. Cell lysates were prepared for immunoblotting and the blots were probed with the indicated antibodies. All immunoblot samples were compared side by side on the same gel; the vertical dashed lines are drawn to simplify comparison. **B-C.** RPE-1 cells were treated with PBCA at the indicated concentrations for 1 h in the absence or presence of BafA1 (100 nM) during autophagy activation by Torin1 (50 nM). Cell lysates were prepared for immunoblotting and the blots were probed with the indicated antibodies. The relative amounts of GABARAP-II were quantified, and the graph shows mean values ± SEM from three independent experiments (C). *, $p < 0.05$.

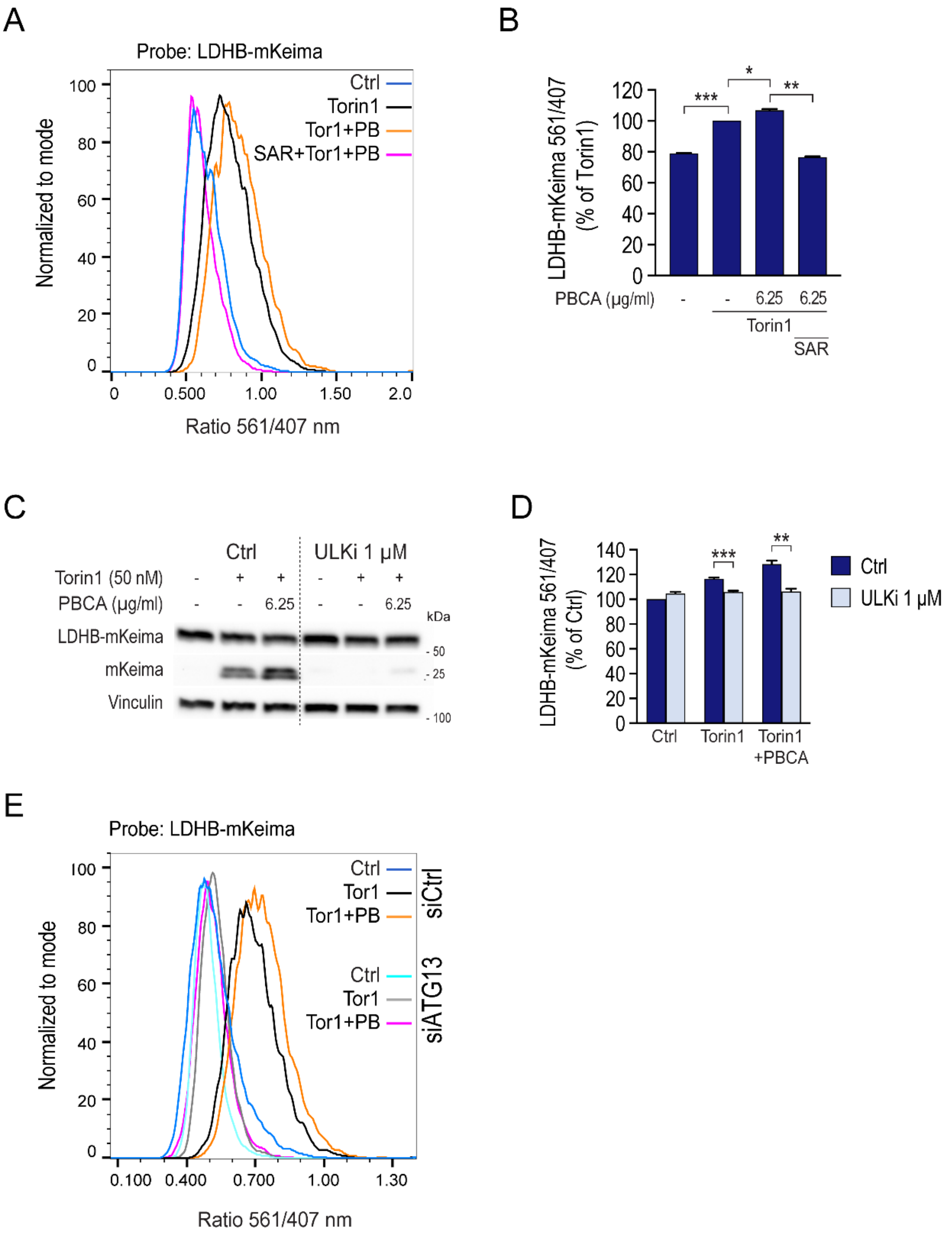

Supplementary Figure S8. The PBCA-mediated potentiation of Torin1-induced autophagy is abolished by SAR405, inhibition of ULK1/2 activity, or depletion of ATG13. A-B. After 48 h of induction with doxycycline (100 ng/ml), the LDHB-mKeima cells were washed twice and treated with Torin1 (50 nM) for 4 h in the absence or presence of PBCA (6.25 µg/ml) and SAR405 (10 µM). The cells were detached and subjected to flow cytometry. The histograms of

the 561/407 nm ratio are shown in A, whereas the median values of the mKeima ratio normalized to cells treated with Torin1 alone are shown in B. **C-D.** RPE-1 cells with inducible expression of LDHB-mKeima were induced for 2 d, washed, and treated with Torin1 (50 nM) and PBCA (6.25 μg/ml) in the absence or presence of the ULK1/2 inhibitor MRT68921 (ULKi) for 4 h. Cell lysates were prepared for immunoblotting (C) or cells were detached for flow cytometry (D). **E.** LDHB-mKeima cells were induced by doxycycline (100 ng/ml) and transfected with either non-targeting control siRNA (siCtrl) or an siRNA targeting ATG13 (siATG13). 48 h later the cells were washed twice and treated with Torin1 (50 nM) in the absence or presence of PBCA (6.25 μg/ml) for 4 h. The cells were detached and subjected to flow cytometry. The histograms of the 561/407 nm ratio for the cell population in each sample are shown. All graphs show mean values ± SEM quantified from at least three independent experiments. *, $p < 0.05$; **, $p < 0.01$; ***, $p < 0.001$.

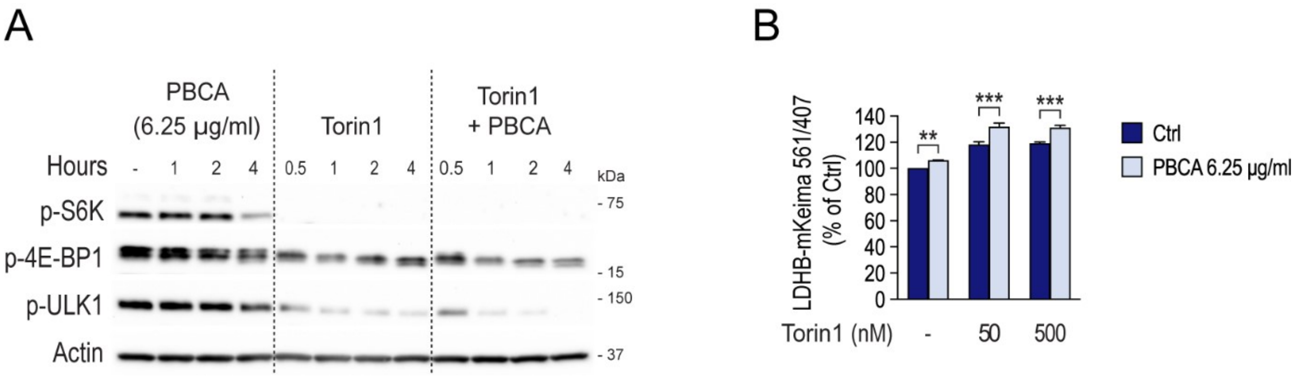

Supplementary Figure S9. PBCA-mediated stimulation of autophagy is mTORC1-independent. A. RPE-1 cells were treated with PBCA (6.25 µg/ml) for the indicated time points in the absence or presence of Torin1 (50 nM). Cell lysates were prepared for immunoblotting and the blots were probed with the indicated antibodies. All immunoblot samples were compared side by side on the same gel; the vertical dashed lines are drawn to simplify comparison. **B.** After 48 h of induction with doxycycline (100 ng/ml), LDHB-mKeima cells were washed twice and subsequently treated with the indicated concentrations of Torin1 for 4 h in the absence or presence of PBCA (6.25 µg/ml). Cells were detached and subjected to flow cytometry analysis. The graph shows mean values ± SEM quantified from at least three independent experiments. **, $p < 0.01$; ***, $p < 0.001$.

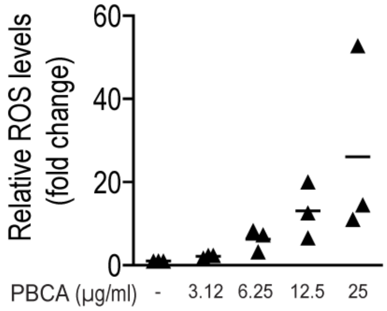

Supplementary Figure S10. Treatment with PBCA leads to generation of ROS. **C**ellular ROS level was assessed by CM-H_2DCFDA fluorescence after treatment of RPE-1 cells with PBCA at the indicated concentrations for 4 h.

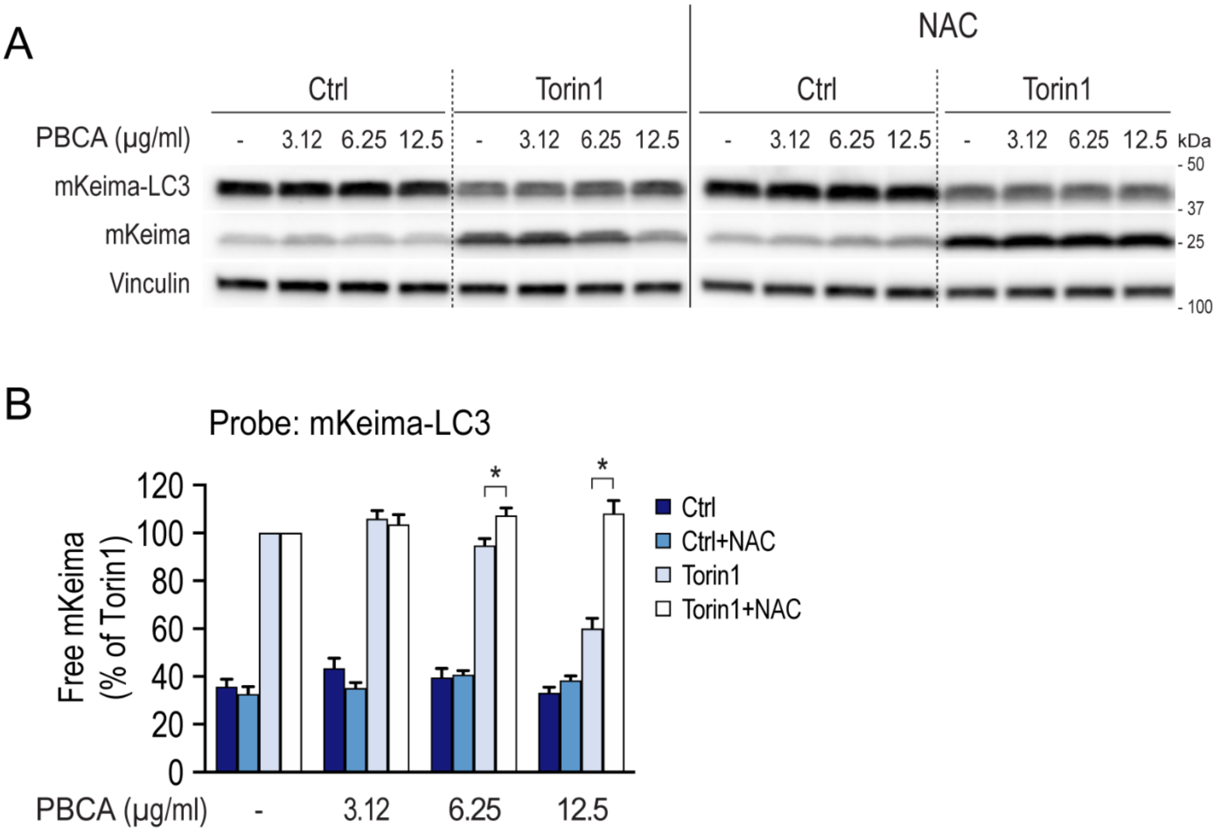

Supplementary Figure S11. NAC prevents PBCA-mediated inhibition of Torin1-induced mKeima-LC3 degradation. After 24 h of induction with doxycycline (100 ng/ml) mKeima-LC3 RPE-1 cells were washed twice and treated with NAC (3 mM) for 1 h. Subsequently, the cells were treated with Torin1 (50 nM) for 4 h in the absence or presence of the indicated concentrations of PBCA. Cell lysates were prepared for immunoblotting and the blots were probed with the indicated antibodies (A). The vertical dashed lines are drawn to simplify comparison and the vertical solid line indicates two separate blots performed in parallel. The relative amounts of free mKeima were quantified (B). The graph shows mean values ± SEM quantified from at least three independent experiments. *, $p < 0.05$.

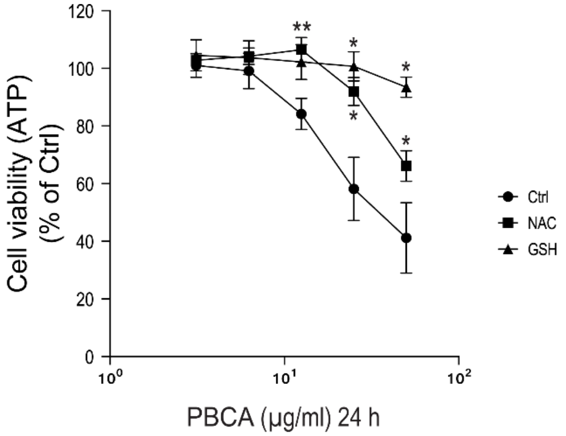

Supplementary Figure S12. Treatment with NAC or GSH reduces the cytotoxicity induced by long-term PBCA incubation. Cell viability assessed by ATP levels of RPE-1 cells treated with PBCA at increasing concentrations for 24 h in the absence or presence of either GSH (10 mM) or NAC (3 mM). The curves show mean values ± SEM quantified from at least three independent experiments. The asterisks denote the statistical significances compared to PBCA-treated cells without antioxidants present. *, $p < 0.05$; **, $p < 0.01$.

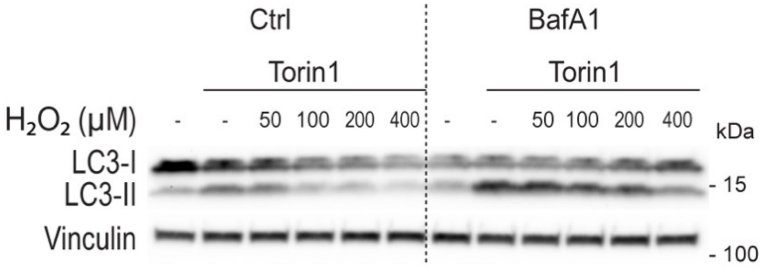

Supplementary Figure S13. Treatment with H_2O_2 reduces Torin1-induced LC3 flux. RPE-1 cells were treated with H_2O_2 at the indicated concentrations for 1 h in the absence or presence of BafA1 (100 nM) during autophagy activation by Torin1 (50 nM). Cell lysates were prepared for immunoblotting and the blots were probed with the indicated antibodies.

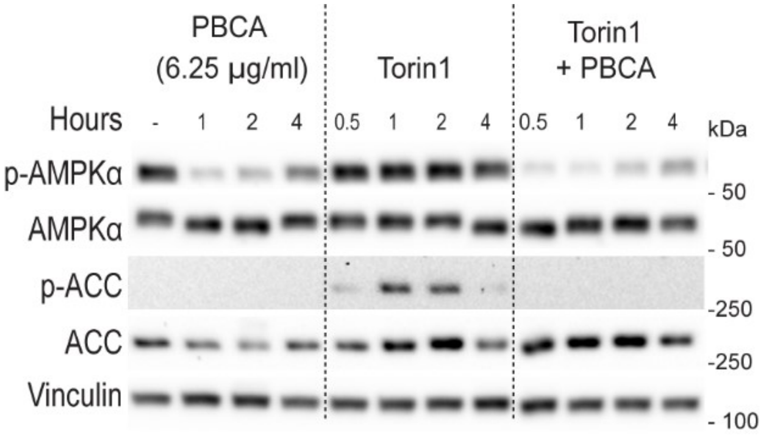

Supplementary Figure S14. Treatment with PBCA inactivates AMPK phosphorylation and downstream signalling. RPE-1 cells were treated with PBCA (6.25 μg/ml) for the indicated time points in the absence or presence of Torin1 (50 nM). Cell lysates were prepared for immunoblotting and the blots were probed with the indicated antibodies. All immunoblot samples were compared side by side on the same gel; the vertical dashed lines are drawn to simplify comparison.

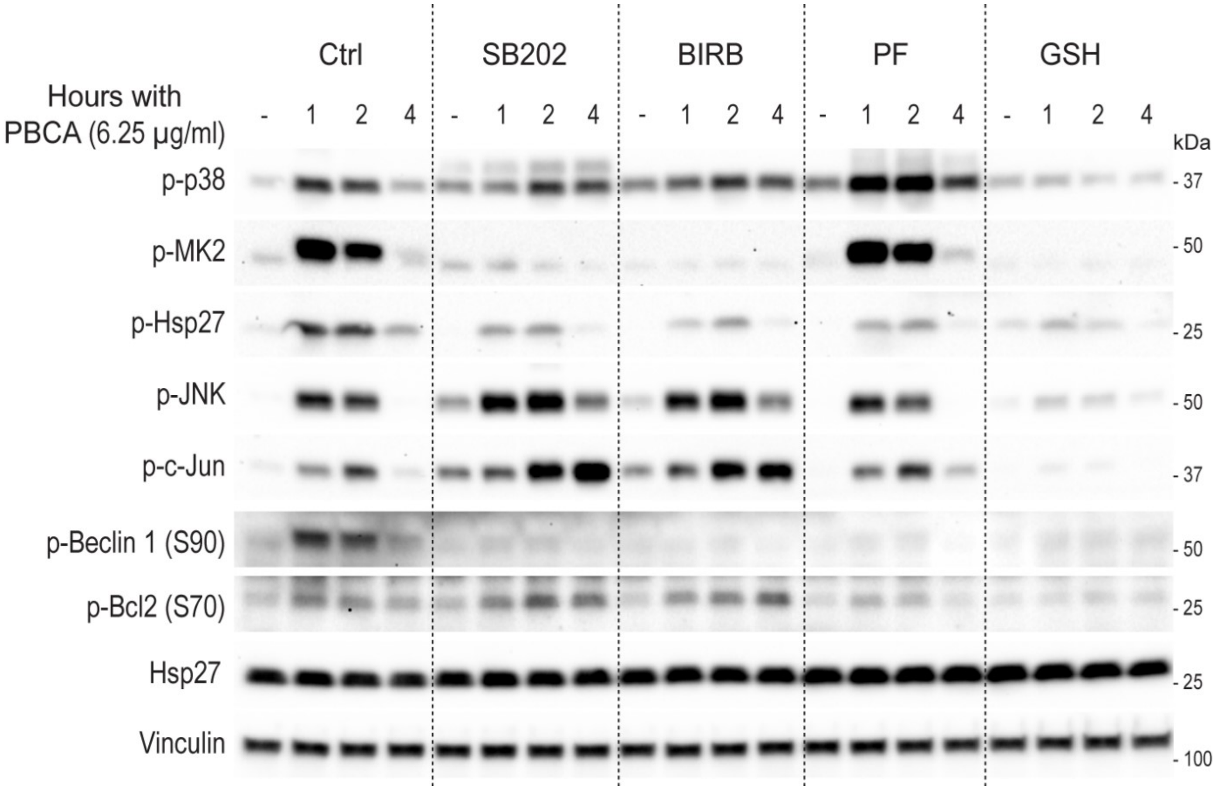

Supplementary Figure S15. Verification of the specificity of the p38α/β inhibitor SB202190, the pan-p38 inhibitor BIRB 796, and the MK2 inhibitor PF-3644022. RPE-1 cells were pre-incubated with SB202190 (SB202, 3 µM, 30 min), BIRB 796 (BIRB, 10 µM, 1.5 h), PF-3644022 (PF, 2.5 µM, 30 min), or GSH (10 mM, 1 h) before treatment with PBCA (6.25 µg/ml) for the indicated time points. Cell lysates were prepared for immunoblotting and the blots were probed with the indicated antibodies. All immunoblot samples were compared side by side on the same gel; the vertical dashed lines are drawn to simplify comparison.

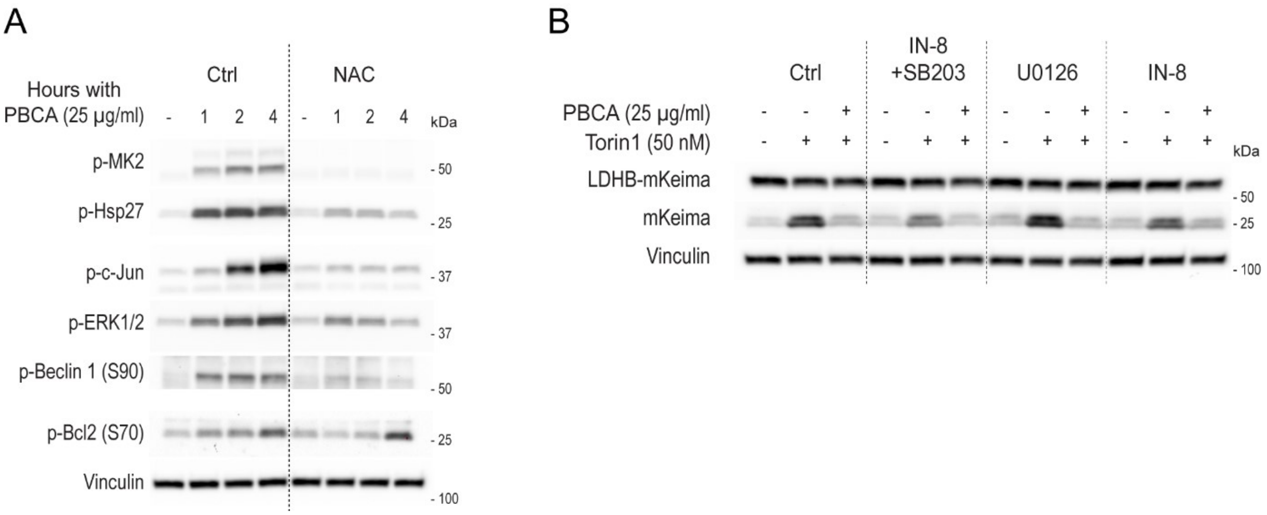

Supplementary Figure S16. High concentration of PBCA induces ROS-dependent, prolonged activation of signalling downstream of p38 and JNK, but this signalling is not responsible for PBCA-mediated inhibition of LDHB-mKeima processing. A. RPE-1 cells were pre-incubated with NAC (3 mM) for 1 h before treatment with PBCA (25 µg/ml) for the indicated time points. Cell lysates were prepared for immunoblotting and the blots were probed with the indicated antibodies. **B.** LDHB-mKeima RPE-1 cells were induced with doxycycline (100 ng/ml) for 2 d, washed twice and then pre-treated with JNK-IN-8 (IN-8, 3 µM, 30 min), SB203580 (SB203, 3 µM, 30 min), or U0126 (10 µM, 1.5 h). Subsequently, the cells were treated with Torin1 (50 nM) for 4 h in the absence or presence of PBCA (25 µg/ml). Cell lysates were prepared for immunoblotting and the blots were probed with the indicated antibodies. All immunoblot samples were compared side by side on the same gel; the vertical dashed lines are drawn to simplify comparison.

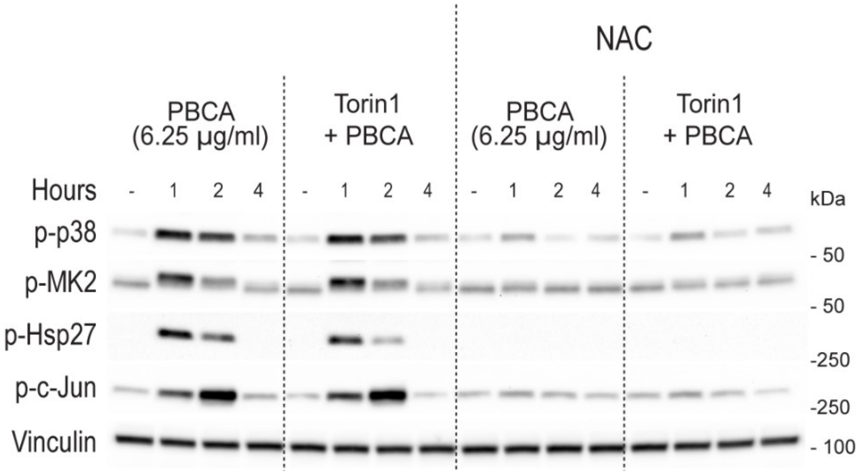

Supplementary Figure S17. PBCA-mediated activation of signalling downstream of p38 and JNK is unaltered in the presence of Torin1, and abolished by NAC. RPE-1 cells were pre-incubated with NAC (3 mM) for 1 h before treatment with PBCA (6.25 µg/ml) for the indicated time points in the absence or presence of Torin1 (50 nM). Cell lysates were prepared for immunoblotting and the blots were probed with the indicated antibodies. All immunoblot samples were compared side by side on the same gel; the vertical dashed lines are drawn to simplify comparison.

References

1. An H, Harper JW. Systematic analysis of ribophagy in human cells reveals bystander flux during selective autophagy. Nat Cell Biol. 2018;20(2):135-143.
2. Szwed M, Sønstevold T, Øverbye A, et al. Small variations in nanoparticle structure dictate differential cellular stress responses and mode of cell death. Nanotoxicology. 2019;13(6):761-782.
